# Associations between host gene expression, the mucosal microbiome, and clinical outcome in the pelvic pouch of patients with inflammatory bowel disease

**DOI:** 10.1186/s13059-015-0637-x

**Published:** 2015-04-08

**Authors:** Xochitl C Morgan, Boyko Kabakchiev, Levi Waldron, Andrea D Tyler, Timothy L Tickle, Raquel Milgrom, Joanne M Stempak, Dirk Gevers, Ramnik J Xavier, Mark S Silverberg, Curtis Huttenhower

**Affiliations:** Department of Biostatistics, Harvard T. H. Chan School of Public Health, 655 Huntington Ave, Boston, MA 02115 USA; The Broad Institute of MIT and Harvard, 415 Main St, Cambridge, MA 02142 USA; Mount Sinai Hospital, Zane Cohen Centre for Digestive Diseases, University of Toronto, 600 University Ave, Toronto, ON M5G 1X5 Canada; City University of New York School of Public Health, Hunter College, 2180 3rd Ave Rm 538, New York, NY 10035-4003 USA

## Abstract

**Background:**

Pouchitis is common after ileal pouch-anal anastomosis (IPAA) surgery for ulcerative colitis (UC). Similar to inflammatory bowel disease (IBD), both host genetics and the microbiota are implicated in its pathogenesis. We use the IPAA model of IBD to associate mucosal host gene expression with mucosal microbiomes and clinical outcomes. We analyze host transcriptomic data and 16S rRNA gene sequencing data from paired biopsies from IPAA patients with UC and familial adenomatous polyposis. To achieve power for a genome-wide microbiome-transcriptome association study, we use principal component analysis for transcript and clade reduction, and identify significant co-variation between clades and transcripts.

**Results:**

Host transcripts co-vary primarily with biopsy location and inflammation, while microbes co-vary primarily with antibiotic use. Transcript-microbe associations are surprisingly modest, but the most strongly microbially-associated host transcript pattern is enriched for complement cascade genes and for the interleukin-12 pathway. Activation of these host processes is inversely correlated with *Sutterella*, *Akkermansia*, *Bifidobacteria*, and *Roseburia* abundance, and positively correlated with *Escherichia* abundance.

**Conclusions:**

This study quantifies the effects of inflammation, antibiotic use, and biopsy location upon the microbiome and host transcriptome during pouchitis. Understanding these effects is essential for basic biological insights as well as for well-designed and adequately-powered studies. Additionally, our study provides a method for profiling host-microbe interactions with appropriate statistical power using high-throughput sequencing, and suggests that cross-sectional changes in gut epithelial transcription are not a major component of the host-microbiome regulatory interface during pouchitis.

**Electronic supplementary material:**

The online version of this article (doi:10.1186/s13059-015-0637-x) contains supplementary material, which is available to authorized users.

## Background

Between 10% and 35% of ulcerative colitis (UC) patients ultimately undergo colectomy with subsequent ileal pouch-anal anastomosis (IPAA) or ‘J-pouch’ construction [1]. Approximately half of patients who undergo IPAA due to UC will have at least one episode of pouchitis, or inflammation of the ileal pouch. In up to 20% of these patients, pouchitis becomes chronic and can lead to pouch failure [1,2]. IPAA is also performed for patients with familial adenomatous polyposis (FAP), but pouchitis is extremely rare in this group [3]. While FAP is associated almost exclusively with defects in the adenomatous polyposis coli gene, UC is associated with polymorphisms in more than 160 IBD-associated genes, including 23 that are UC-specific [4], indicating that complex host genetics may play a crucial role in the onset of pouchitis. The gut microbiome is also highly influential in both IBD and pouchitis [5-9]; most episodes of acute pouchitis can be treated with a course of antibiotics and may be prevented by probiotic use [3] but antibiotics have shown somewhat mixed results in their efficacy for treating Crohn’s disease (CD) and UC [10,11]. This combination of physiological similarities and genetic differences makes pouchitis an appropriate model in which to examine the interplay of inflammatory disease, gut microbes, and host gene activity [12].

While it is known that both host genetics and the microbiome influence the development of pouchitis, precisely how they interact is less well-understood. Following IPAA surgery, the mucosal structure of the J-pouch becomes more colon-like; villous structures become more shallow, mucin expression changes [13], and the microbial community becomes functionally more similar to a colonic community [14]. It is unclear, however, whether pouchitis is a recurrence of UC that manifests as the host postoperative ileum and microbiome collectively become more colon-like, or a unique disease with characteristics of both CD and UC. However, by simultaneously measuring the microbiome and host transcriptome, we may begin to understand the relationships between microbiota, host, and disease pathogenesis.

To gain insight into these host-microbe interactions in the epithelial mucosa, we have collected paired host transcriptome and microbial metagenome data from a large J-pouch cohort, allowing us to measure whether elevated or depleted host epithelial transcripts are associated with specific microbial clades. While other studies have applied sequencing to the IPAA microbiome, these had small numbers of patients [14,15] or did not concurrently examine host gene expression [9,16]. Likewise, few studies have comprehensively measured the IPAA host microbiome and transcriptome [17,18]. To the best of our knowledge, ours is the first study to examine both. In this study we use the IPAA model to study the relationship between the IPAA microbiome and host gene expression. We have recruited a large population of patients having undergone IPAA at Mount Sinai Hospital, a large, tertiary care referral center in Toronto, Canada. These subjects were identified as part of a wider study investigating the etiology of pouch complications. Thus, this cohort had a wide variety of both molecular and clinical data available for analysis, including detailed information regarding postsurgical outcomes.

The gut microbiome in this cohort was most affected by inter-individual differences in antibiotic usage, while epithelial transcription was more strongly influenced by tissue location (pouch vs. pre-pouch ileum). A very small proportion of microbial or transcriptional variation was explained by host-microbe correspondences, in that associations of the host transcriptome with the microbiome were relatively modest in comparison to other effects. We developed a dimensionality reduction process to ensure appropriate statistical power for testing these associations, due to the large number of transcripts and operational taxonomic units (OTUs) observed relative to number of samples, comparable to the analysis methods necessary for eQTL or similar studies [19-21]. After employing both supervised and unsupervised data reduction methods, we used multivariate linear modeling to identify significant associations between microbes, transcripts, and environment, as described above, as well as between the overall patterns of host transcription and microbial composition. These were primarily related to level of host inflammation as, for example, the most microbially-associated host transcript pattern (gPC9) was enriched for complement and IL-12 components in GSEA analysis (Additional file 1C). Finally, discriminant modeling of pouchitis outcome by linear discriminant analysis proved to be ineffective using either microbial composition, transcriptional activity, or both, in antibiotic-free samples.

## Results

### A multivariate model for co-analysis of host epithelial tissue gene expression, gut tissue-associated microbiome structure, and cohort characteristics and clinical phenotype

**Figure 1:**
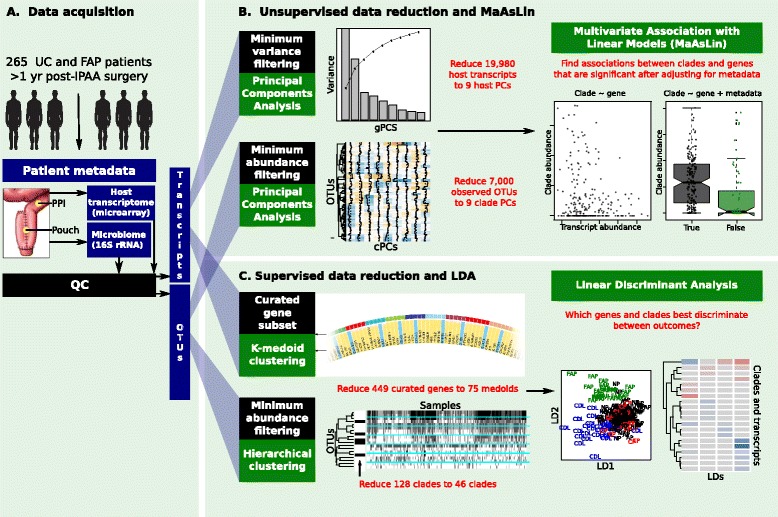
Overview of data analysis. **(A)** Data were acquired from a cohort of 265 UC and FAP patients who had IPAA surgery at least 1 year previously. Biopsies were collected from each patient from both the pre-pouch ileum and j-pouch. The host transcriptome was profiled using cDNA microarrays, and the microbiome was profiled by sequencing the V4 region of the 16S gene. Data were then subjected to unsupervised reduction and linear modeling (B), and to supervised reduction and linear discriminant analysis (C). **(B)** After quality control, data dimensionality was reduced to maximize statistical power prior to linear modeling. After filtering low-variance transcripts, principal component analysis was used to create nine gene principal components (gPCs) to account for 50% of the variance in the transcriptome data. OTUs were filtered for minimum abundance and for presence in at least three samples. PCA was then used to create nine clade principal components (cPCs) explaining 50% of the variance in OTU data. Multivariate association with linear modeling was then used to test for associations between clades and transcripts that were significant after adjusting for metadata (inflammation, antibiotic use, and outcome). **(C)** In an alternative data reduction approach, a list of 449 genes was curated from IBD genome-wide association studies [4] and host genes that physically interact with bacteria [22]. The expression profiles of these 449 genes were further reduced by k-medoid clustering into 75 medoids, each representing a cluster of genes with similar expression profiles. Abundant microbial clades were hierarchically clustered, and one representative from each cluster was chosen. Linear discriminant analysis was used to measure which genes and clades were most discriminant between clinical outcomes. (See also Additional file 1, Additional file 2, and Additional file 3A to C).

In order to better understand the relationships between the host and microbiome after IPAA surgery, we measured host gene expression by microarray [17] and the microbial community using the 16S rRNA gene [9] (referred to hereafter as 16S) in a large, metadata-rich, cross-sectional cohort. The cohort consisted of 265 patients (51% women) aged between 18 and 78 years (median age, 48 years; Table 1). Patients who had surgical management of UC or FAP were included, and all patients had IPAA surgery at least 1 year prior to biopsy collection for this study. Patients were classified as FAP (Familial Adenomatous Polyposis), No Pouchitis, Acute Pouchitis, Chronic Pouchitis, or Crohn’s Disease-Like Inflammation (see Methods for criteria). Most patients were biopsied in both the pouch (P) and in the pre-pouch ileum (PPI). After quality control, there was host gene expression and microbiome data obtained by microarray and 16S analysis from a total of 255 samples representing 204 individuals (Methods, Figure 1); these comprised 196 PPI samples and 59 pouch samples.

**Table 1:** **Demographic and clinical characteristics of IPAA cohort**

	**Patients cohort (** ***n*** **= 265)**
Age at recruitment, years (mean, range)	47 (18–76)
Gender (% female)	135 (50.5)
Time since ileostomy closure (mean years, range)	12 (1–40)
Smoking (% at recruitment)	24 (9.2)
Antibiotic use previous month (%)	78 (29.4)
Distribution of patients in phenotypic outcome groups, number (%)	
FAP	32 (12)
NP	72 (27)
CP	27 (10)
CDL	34 (13)
AP	69 (26)

All recruited patients had IPAA surgery >1 year prior to recruitment except for two, whose previous diagnoses were pouchitis and FAP, respectively.

### Between-tissue variation is high for host gene expression but low for the microbiome

Previous studies in a subset of this cohort demonstrated that there were few differences in the microbiome between pouch and PPI samples [9], yet a great deal of variability was observed between these sites in the tissue transcriptome [9,17]. As expected, we observed that the Bray-Curtis distance for microbial profiles between locations was much lower than between individuals, indicating that the microbial profiles of pouch and PPI were similar (Additional file 2). In contrast, the within-site variation in gene expression based on Pearson correlation was nearly as great as the between-individual variation, indicating that tissue location (pouch vs. PPI) was a large source of transcriptional variation.

### Dimensionality reduction for well-powered multi-omic data integration in a human cohort

In order to improve power to associate microbial composition with host transcriptional activity, we reduced the dimensionality of both host and microbial features. We first calculated that given a true covariance of 0.5 in the data between microbial abundance and gene expression, it would be possible to perform a maximum of 10^4^ pairwise tests and retain 90% power and an alpha equal to 0.05 using Bonferroni correction (Additional file 1A). Thus, it was necessary to reduce 19,908 host transcripts and 6,999 observed OTUs to 10^4^ tests, or approximately 100 transcripts and 100 clades of interest.

We pursued several broad strategies to achieve this goal. First, we limited our analysis of OTUs to only those that were both present in multiple individuals and abundant, with mean abundance >0.005 (see Methods). Second, we employed both further unsupervised and supervised strategies for data reduction prior to our downstream analysis, which included multivariate linear modeling (which aimed to associate microbes with host transcripts) and linear discriminant analysis (which aimed to determine which microbes and transcripts were most discriminant of clinical outcome; Figure 1).

For unsupervised dimensionality reduction of microbial data, after OTUs were abundance-filtered, we applied a variance-stabilizing arcsin-square transformation, then used principal component analysis to reduce these filtered, abundant clades to nine clade principal components (cPCs) that explained 50% of observed variance (Figure 1). The loadings of each cPC represent a pattern of highly correlated microbial abundances (Additional file 1D; Additional file 3A, B). For supervised clade reduction, we further reduced the filtered list of microbial clades by hierarchically clustering it, then selecting the lowest-mean-abundance representative from each cluster. This had the practical effect of removing redundant higher-order taxonomic clades from the list of taxa, and it reduced the total number of microbial clades to 45 (Figure 1).

Supervised transcript reduction aimed to focus upon host genes of particular prior interest, specifically those that had been previously implicated in IBD, pouchitis, or host-microbe interactions. Thus, we curated a set of 174 IBD-associated genes [4], 272 bacterially-interacting genes [22], and 12 pouchitis-related genes from the literature (Methods), and the expression profiles of these genes were clustered into 75 gene medoids, each of which represented one or several similarly-expressed genes (Additional file 3C). For unsupervised reduction of transcripts, we first filtered all host transcripts to remove the two quantiles of genes whose expression varied the least across all subjects. Next, we used principal component analysis to reduce the remaining 11,945 host transcripts to a collection of nine transcript principal components (gPCs) explaining 50% of all observed variance. Again, the loadings of each principal component represent a pattern of highly correlated transcript abundances.

Through these data reduction methods, we transformed 19,908 host transcripts and 6,999 observed OTUs into a total of 138 features. There were nine transcript principal components and nine clade principal components, which had been chosen in an unsupervised manner. In addition, there were 75 gene medoids and 45 clades, which had been selected in a more supervised manner. These 138 features were used for subsequent analysis.

### Tissue location and antibiotic use induce the greatest changes in host gene expression and microbiome composition, respectively

**Figure 2:**
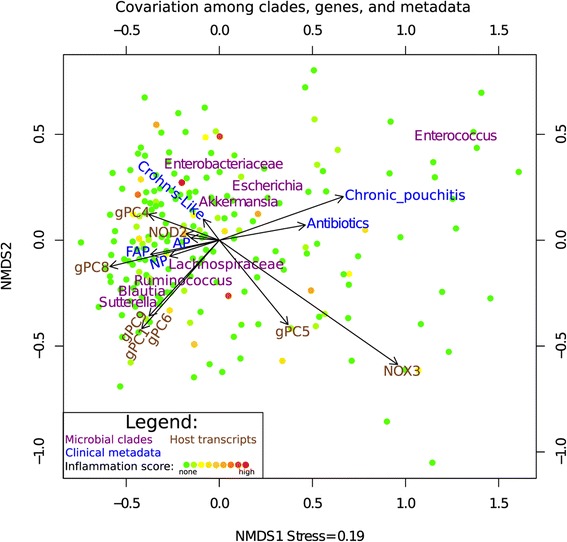
Biplot of clades, genes, and study metadata. Non-metric multidimensional scaling (NMDS) of clade abundances was used to position samples and show samples relatively enriched in specific clades (purple). Arrows represent host transcripts (brown) and metadata (blue), which include antibiotic use and clinical outcome. Arrow coordinates are determined by averaging the coordinates of each sample containing a specific metadata, and show the central tendency of the metadata. Samples are color-coded according to inflammation, which ranges from none (green) to high (red). This figure was created with PPI-only samples.

After initial gene and clade reduction, in order to provide an initial visualization of the relationships between gPCs, cPCs, medoids of interest, inflammation, antibiotic use, and clinical outcome, we generated a biplot using the Breadcrumbs package ([23], Figure 2). The strongest data separation effect corresponded to antibiotic use, which was highly correlated both with the chronic pouchitis phenotype and with abundant *Enterococcus*, which is frequently resistant to both metronidazole and ciprofloxacin [24,25]. In contrast, high expression of gPC8 was inversely correlated with antibiotic use (Figure 2). Crohn’s disease-like inflammation was modestly associated with increased Enterobacteriaceae, while high expression of gPC9 was associated with more abundant *Sutterella* and beneficial Clostridia, including *Ruminococcus* and *Blautia*. The transcript patterns gPC1, gPC9, and gPC6 were most closely associated with FAP or no pouchitis (Figure 2).

Next, we quantified the proportions of the microbiome and total host transcriptome that were affected by tissue location (pouch vs. PPI), clinical outcome, antibiotic use, and inflammation, using univariate association tests of each transcript and each clade with the metadata. The extent of shift is summarized as the percentage of transcriptome or microbiome features differentially expressed at FDR <0.05 (Table 2; Additional file 3D to I). As previously shown [17], host transcripts were most strongly associated with location, followed by inflammation, with little or no association with antibiotic use. When we subjected the differentially-expressed transcripts between pouch and PPI to gene ontology enrichment analysis by GOrilla [26], the transcript category most significantly affected was transporters (Additional file 4). The transcriptional differences between pouch and PPI and are described in detail by Kabakchiev *et al.* [17]. In contrast, differential expression of microbial clades was strongly associated with antibiotics, but very few clades were differentially expressed in association with inflammation or tissue type (Table 2; Additional file 3D, E, I). Large differences in microbial significance (for example, 41% of microbes in PPI significantly affected by antibiotics vs. 2% in pouch) are likely due to the large discrepancy in number of pouch vs. PPI samples (59 vs. 196 samples) (Additional file 5B), resulting in fewer pouch taxa reaching significance.

**Table 2:** **The effects of inflammation, antibiotics, outcome, and sample location on the transcriptome and microbiome**

	**Transcriptome**		**Microbiome**	
	**PPI**	**Pouch**	**PPI**	**Pouch**
Inflammation score	19%	22%	0%	5%
Antibiotics	0%	0%	41%	2%
Outcome	0%	15%	3%	3%
Location	45%		1%	

This table shows the percentages of the transcriptome and microbiome that are differentially expressed (FDR <0.05) with respect to inflammation score (continuous scale 0–12), antibiotics usage (yes/no), outcome (AP, NP, CP, CDL), and sampling location (pouch or pre-pouch ileum). The microbiome is extensively shifted by antibiotics usage with minor shifts by outcome and location, whereas the host transcriptome is extensively shifted between locations and by inflammation. See also (Additional file 4, Additional file 3D to I).

**Figure 3:**
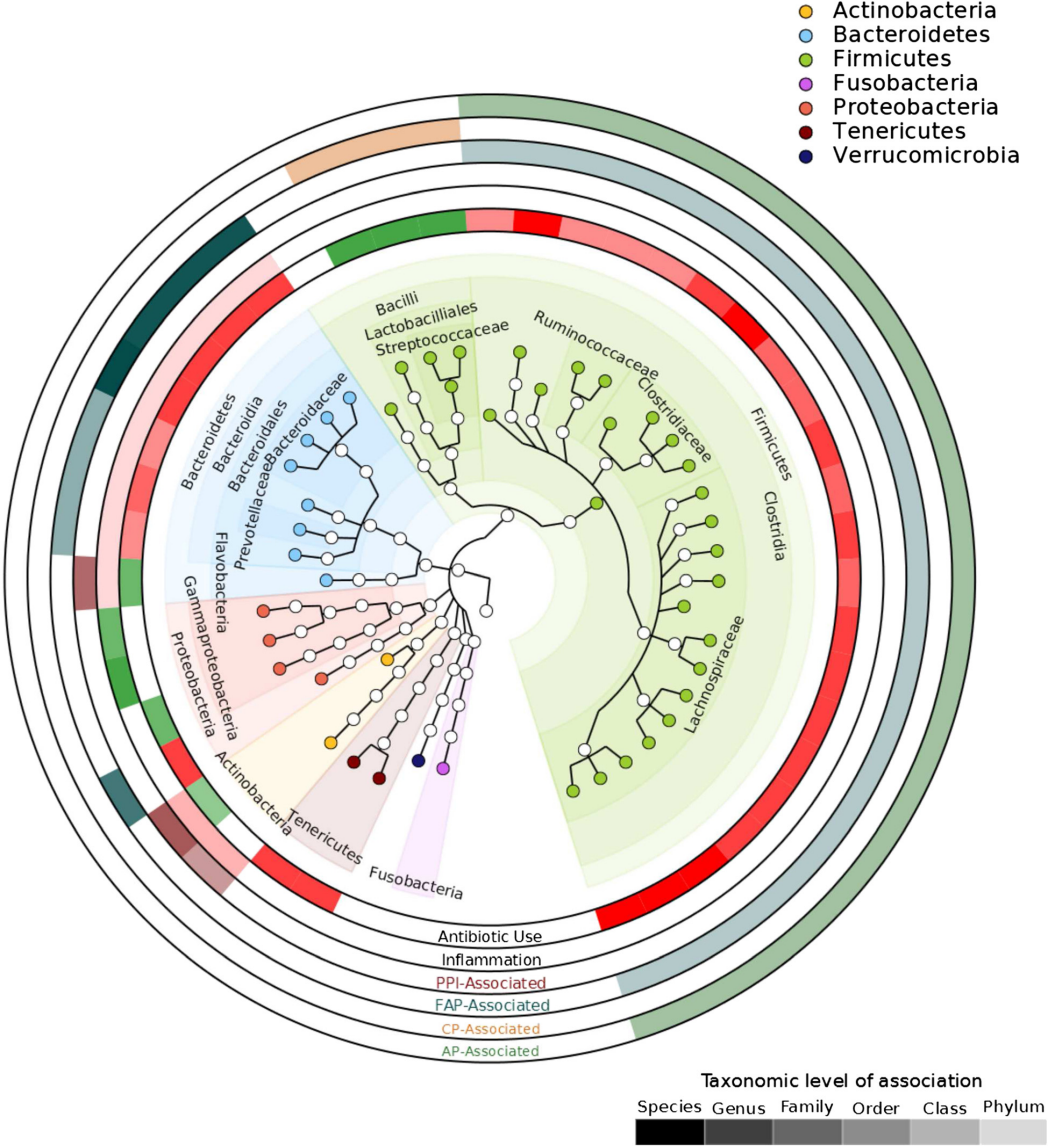
The relationship between clades and metadata in univariate analysis. The major metadata in the cohort were antibiotic use, inflammation, tissue (pouch or PPI), and outcome (AP, NP, CP, FAP, or CDL). Univariate linear discriminant analysis effect size analysis was performed on each of these variables. Antibiotic use was associated the greatest number of perturbations in the microbiome, causing broad decreases in the Clostridia, Bacteroides, Tenericutes, and Betaproteobacteria, and increases in the Lactobacilliales, Actinobacteria, and Gammaproteobacteria. Because the antibiotic effect size was very large and affected most clades, LDA effects for inflammation (ring 2), tissue types (ring 3), and outcomes (rings 4, 5, and 6) were calculated after stratifying for antibiotic use. Color intensity of ring corresponds to the taxonomic level at which the LDA effect is significant (*P* <0.05), from phylum (least intense) to genus (most intense).

In order to further investigate the effects of antibiotics, tissue location, clinical outcome, and inflammation upon specific microbial clades, and to visualize the phylogenetic relationships of these affected clades, we conducted an independent univariate analysis of with LEfSe [27], which is shown in Figure 3. As antibiotic use was the largest effect, LDA effects for inflammation, tissue, and clinical outcome were stratified by antibiotic use (Figure 3). There was a broad decrease in the abundance of Bacteroides, Firmicutes, and Tenericutes that was associated with antibiotic use. There was an antibiotic-associated increase in the abundance of Bacilli and gammaproteobacteria that was spurred primarily by *Enterococcus* and Pasteurellaceae. Although *Enterococcus* was strongly associated (*P* <0.05) with the chronic pouchitis phenotype in univariate analysis, it was not significant when stratified by antibiotic use because it was not elevated in antibiotic-free samples (Additional file 5C). *Escherichia* were positively associated with inflammation, while the Actinobacteria were negatively associated. The genus *Sutterella* and generally higher levels of Bacteroidetes were strongly associated with the outcome FAP even after accounting for antibiotic use. Actinomycetales and Flavobacteria were weakly associated with the PPI. However, antibiotic effects on the microbiota were much stronger and more widespread than effects due to tissue, inflammation, or clinical outcome.

### Host gene expression is not a major determinant of pouch microbial community composition

**Figure 4:**
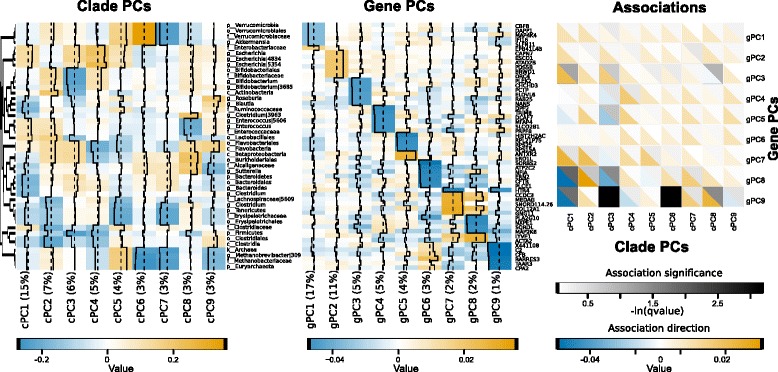
Results of multivariate linear modeling. Principal component analysis was used to reduce the data into nine gPCs and cPCs that explained 50% of total transcriptional and microbial variation. The top six loadings for each cPC (left) and cPC (middle) are shown; orange and blue indicate increases or decreases in expression, respectively. (Right) MaAsLin [5,28] was used for multivariate linear analysis of associations between cPCs and gPCs while controlling for the effects of inflammation, tissue location, and antibiotic use. Black/gray scale corresponds to the significance of the association, while blue / orange corresponds to the direction. See also Additional file 5.

Following data reduction, in order to measure gene-clade associations, we used MaAsLin [5,28] to apply a multivariate linear model which controlled for the effects of antibiotic use and inflammation (see Methods). Although pouch and PPI microbiome profiles were highly similar within the same individual, pouch-PPI transcriptomes were not. Under these circumstances, we did not expect any gain in power for detecting microbiome-transcriptome associations from the addition of PPI samples by inclusion of a random effect for individual to the linear model. Thus, we excluded the relatively small number of paired pouch samples from association testing (Figure 1B). The supervised (curated gene) and unsupervised (gPC/cPC) gene lists were run through MaAsLin independently; only the unsupervised results were significant (Figure 4).

The only gPCs significantly associated with cPCs were gPC8 and gPC9 (q <0.25). The top loadings of gPC9 reflected reduced expression of the complement cascade (CFI, C2, and CFB), interferon regulatory factor 1, interferon-induced guanylate binding protein, and the leukocyte chemotaxis factor CCL2, indicating that high expression of gPC9 may correspond to a lower overall state of inflammation. Indeed, when samples were stratified by clinical outcome, gPC9 was lowest-expressed in patients with Crohn’s disease-like inflammation, and highest-expressed in patients with FAP (Additional file 5A). The top loadings of gPC8 included reduced expression of the lipopolysaccharide-activated p38 MAP kinase Map2K6 and of PLA2G10, which is involved in calcium and fat-mediated inflammatory signaling and eicosanoid release; thus, gPC8 may also be related to inflammation. However, when stratified by antibiotic use or clinical outcome, gPC8 was less differentially expressed than gPC9 (Additional file 5A, Additional file 3B).

A total of four clade cPCs were associated with gPC8 and gPC9: cPC1, cPC3, cPC6, and cPC8. The loadings of cPC1, which accounted for 15% of the observed variance, show several features apparently corresponding to antibiotic use: increased Enterobacteriaceae abundance, a broad decrease in Bacteroides and Firmicutes, and among the highest abundance of Enterococcus (Figure 4). Indeed, cPC1 was also more abundant in patients who had been taking antibiotics (Additional file 5A). cPC3 featured the lowest levels of *Bifidobacterium*. cPC1 and cPC3 were negatively associated with gPC8 and gPC9; thus, these patterns indicate that an antibiotic-signature microbiome was associated with higher potentially inflammatory gene expression. However, in contrast to cPC1, cPC3 was not differentially abundant when stratified by outcome or antibiotic use (Additional file 5A).

The most remarkable feature of cPC6 loadings was its high abundance of *Akkermansia*, a beneficial mucin-utilizing microbe [29]; cPC6 was also evenly distributed among outcomes and antibiotic use (Additional file 5A). cPC8 loadings were noteworthy for their high abundance of the genus *Sutterella*, and lower abundance of cPC8 was associated with chronic pouchitis and antibiotic use (Additional file 5A). While some studies have associated *Sutterella* with autism [30,31], in our cohort, it was associated with the healthy FAP outcome (Figure 3). A recent study also found that *Sutterella* was decreased in new-onset Crohn’s disease [32].

Together, the linear relationship between host transcripts and microbes was generally modest, representing approximately 25% of total variance, as variation is driven primarily by location and by antibiotic use, respectively. However, these data represent the strongest transcript-microbe associations in the cohort after variation from antibiotic and tissue has been factored out. The strongest relationships we observed appear to be associated with inflammation-associated loadings. Other potential relationships may be better explored with additional samples for more statistical power.

### Using a joint host-microbe model to segregate pouch outcome

It is of great clinical interest to know whether host transcripts, microbes, or some combination thereof can be used to distinguish clinical outcomes. To explore this question, we used linear discriminant analysis (LDA) to identify which combinations of genes and microbes were most able to cross-sectionally segregate clinical outcome in a training set, then assessed accuracy in cross-validation (see Methods). Because antibiotic use was highly asymmetrical across clinical outcomes (Additional file 5B) and highly predictive of the chronic pouchitis outcome, we limited this analysis to those samples without antibiotic use (Additional file 6).

**Figure 5:**
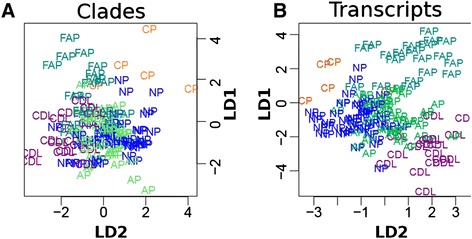
Linear discriminant analysis for clinical outcome. Linear discriminant analysis was used to determine which genes and clades were most discriminant between clinical outcomes after controlling for antibiotic use. All samples with antibiotic use were removed prior to analysis, and an LDA fitting model with leave-one-out cross-validation was used. **(A, B)** The separation of clinical outcomes by LD1 and LD2. See also (Additional file 6).

CDL and CP were best discriminated by this model, particularly with respect to FAP (Figure 5). However, accuracy was low upon cross-validation (mean AUC 0.57 across all outcomes and models, Additional file 6A), primarily due to the model’s lower discrimination of AP and NP outcomes. These represent the extremes of outcome phenotypes in several respects, particularly with respect to inflammation. While this is also true for antibiotic usage (highly prevalent in CDL and rare in FAP), this analysis specifically excluded all samples from antibiotic-treated patients, as these proved to be very well-discriminated using microbial profiles alone. Indeed, when antibiotic-treated samples were included, discrimination accuracy for the CDL (AUC 0.67), CP (AUC 0.88), and FAP (AUC 0.71) outcomes was much higher based solely on models of microbiome profiles (Additional file 6B). When we examined the separation ability of the LDs (Figure 5, Additional file 6C), they were most discriminant between FAP and CDL.

## Discussion

Although this study and many others have observed that the mucosal microbiome is highly variable between any two individuals [33,34], the host mucosal transcriptome appears to be a surprisingly small correlate of this variation in microbial community composition. Here, the transcriptome showed large variation between the pre-pouch ileum and the pouch within the same individual; for example, there were significant differences in the expression of amino acid, heme, and metal ion transporters (Additional file 4). Despite these large transcriptional differences between tissue locations, the microbial community within each individual remained similar between these two environments. It is important to note that our methods would not resolve sub-genus-level differences in the mucosal communities, and that mucosal communities are likely to show less homogeneity over greater biogeographic distances in the GI tract [35]. However, these findings suggest that the composition of an individual’s microbiome in adulthood may not be shaped by local transcriptional activity on a long-term basis, but rather by factors such as initial early life colonization events [36-39] or diet [40] over time spans relevant for disease development. Conversely, inter-individual differences in the microbiome appear not to drive correspondingly large changes in gene expression.

As expected, the largest effect on the microbiome is antibiotic use. Metronidazole, the antibiotic most commonly used to treat pouchitis, kills anaerobic bacteria by damaging their DNA [41], thus profoundly decreasing the populations of Bacteroidetes and Clostridiaceae. The resistance of facultative anaerobes to metronidazole is much more variable; *Gardnerella* is highly susceptible [41], while *Eikenella* is highly resistant [42], and resistance in *Propionibacterium* appears to correlate with the presence of *nim* genes [43]. In our data from the pelvic pouch, the Bacteroidetes and Clostridiaceae appeared to be displaced by facultative anaerobes such as the Lactobacilliales (for example, *Enterococcus* and *Streptoccus*) and gammaproteobacteria (for example, Pasteurellaceae). *Enterococcus* genomes are highly recombinant and remarkable as a reservoir of antibiotic resistance, and thus a public health concern [44]. Their metronidazole resistance is well-known [45-47], and they are becoming increasingly resistant to ciprofloxacin [48-50], which is an antibiotic of choice for pouchitis. Although the antibiotic-resistance profiles of human-associated *Pasteurella* have been much less widely described, a study of swine-associated *Pasteurella* strains found that they were highly resistant to metronidazole (but not quinolones) [51], which is consistent with our observations.

We found in univariate analysis that after accounting for the effects of antibiotic use, pouch inflammation influenced relatively few taxa; specifically, it enriched for *Escherichia*, while there were non-specific inflammation-associated decreases in the class Actinobacteria and in the phylum Bacteroidetes (Figure 3). This is consistent with *Escherichia*’s role as a facultative anaerobe that is frequently enriched in Crohn’s disease [5,52]. Inasmuch as many microbial surveys of CD patients have found no species as consistently overrepresented in IBD as *Escherichia*, and this overrepresentation appears to be a feature of later IBD rather than early IBD [32], it is possible that *Escherichia* is unique among the intestinal microbiota in its ability to thrive in chronic redox stress. It has recently been shown that nitrate respiration in the inflamed host gut is at least one of the mechanisms by which *Escherichia* may gain an advantage [53]. Alternatively, our ability to associate microbes with inflammation may be reduced by perturbations already induced in the microbiome as, for example, by pouch surgery prior to sampling.

The transcript pattern gPC9 demonstrated the broadest range of associations identified between host transcription and microbial community structure. Its individual gene loading components (including complement cascade, immune cell adhesion, p38 MAP kinase genes) were functionally associated with inflammation, but expression of gPC9 itself was not correlated with the clinical inflammatory score (r_s_ = 0.02) (Additional file 5D). There was a slightly greater negative correlation between gPC9 and the abundance of *Escherichia* (r_s_ = −0.29) (Additional file 5E). gPC9 was positively associated with cPC6; the most abundant clade in this cPC was *Akkermansia,* which has previously been associated with improvement of metabolic syndrome and DSS colitis [29,54], as well as increased susceptibility to Salmonella [55]. Taken together, sub-clinical inflammation may thus be inducing a modest but detectable effect on the microbiome detectable in these data and in a corresponding host transcriptional response, even prior to being histologically detectable.

Dimensionality reduction was a key component in making this study possible; as with genome-wide association studies or eQTL associations, naive testing of all possible hypotheses would require an exceptionally large cohort. As this is rarely possible in practice, we used principal component analysis for unsupervised data reduction, and k-medoids clustering of a curated gene list for supervised data reduction. Other recent papers [40,56-58] have employed similar clustering-based data reduction strategies to find signal in relatively small datasets. These results also underscore the importance of designing microbial association studies to include an explicit, up-front power analysis and of having realistic expectations about the effect sizes to be observed; they are likely to be modest effects, similar to GWAS, rather than large effects. Here, for example, the strongest microbe-transcript correlations were approximately 0.2 to 0.3, and it would have been impossible for significant associations to survive correction for multiple hypothesis testing if all genes and clades were simultaneously analyzed. This must be anticipated when planning studies to ensure they are designed with appropriate sample sizes.

Finally, discriminating clinical outcome based on the microbiome and transcriptome was a complex problem intractable to LDA analysis. While chronic pouchitis could be accurately distinguished after the fact based on antibiotic use (Additional file 6), this is not clinically useful. Cross-sectional data may particularly limit the utility of LDA for exploring this problem, given the high degree of between-individual variation in microbiota and the temporal nature of pouchitis and antibiotic use. While it is clearly not feasible to biopsy subjects repeatedly over short periods of time, it would be reasonable to study the relationship between microbiota and onset of chronic pouchitis with longitudinal stool collection. More stable markers, such as SNPs and serum antibodies may also have better utility in classifying postoperative pouch outcomes [2].

## Conclusion

In conclusion, the primary influences upon host gene expression and the microbiome appeared to be distinct by several measures in this cohort. We observed modest associations between groups of host transcripts involved in inflammation and clades such as *Sutterella*, *Akkermansia*, and *Bifidobacterium*, but these were not among the greatest sources of variation in community structure or gene expression. Instead, the former was greatly influenced by pharmaceutical treatments (specifically antibiotics), and the latter by tissue location. Thus, while pouchitis clinical outcomes were well-differentiated by naive linear discriminant analysis, this was due almost exclusively to differences in antibiotic usage among outcomes and may be a problem better-suited to longitudinal data. Although we are able to observe significant host-transcript associations, the effect sizes are modest, indicating that other factors, such as initial host colonization and diet, are also significant influencers of microbial composition. To distinguish these effects, we will need additional data from well-powered studies.

## Methods

### Patient cohort

Patients having undergone proctocolectomy with ileal pouch-anal anastomosis (IPAA) for treatment of UC or FAP at least 1 year prior to enrollment, were recruited at Mount Sinai Hospital (Toronto, Canada). Individuals with a diagnosis of CD were excluded. Patients underwent pouch endoscopy with biopsy, and completed a questionnaire encompassing demographic and clinical elements. Physicians documented the appearance of the pouch using specific evaluation criteria outlined in the pouchitis activity score (PAS). Specifically, to numerically score inflammation, the severity of objective traits was graded (erythema, friability, and ulceration at the time of endoscopy, and polymorphonuclear leukocyte infiltration and ulceration by histology) according to the numeric scale described by Tyler *et al.* [9], and the inflammation score was defined as the sum of these traits. A total inflammation score of 14 was possible, but any score over 3 was considered inflamed. Subjects were classified based on postsurgical phenotypic outcome using a combination of long-term history following surgery and inflammatory activity at the time of pouch endoscopy, as has been previously described [9]: Familial Adenomatous Polyposis (FAP) with no inflammatory complications post-surgery; No Pouchitis (NP) with no previous documented episodes of pouchitis and no evidence of pouchitis at the time of pouchoscopy; Acute Pouchitis (AP) based on historical or current documentation of inflammation of the pouch resolving after a single course of antibiotics; Chronic Pouchitis (CP), including antibiotic-dependent and antibiotic-refractory patients who required either prolonged (>1 month) antibiotic therapy, medical intervention for pouchitis more than three times per year, or the use of second- or third-line medications (5-ASA, steroids, immunomodulators, biologics); or Crohn’s disease-like phenotype (CDL) based on a patient developing an abscess or fistula more than 1 year following ileostomy closure, or inflammation in the afferent limb or proximal small bowel. Subject recruitment and study procedures were approved by and carried out in accordance with the Research Ethics Board of Mount Sinai Hospital (Toronto, Canada), with the following tracking information: 08-0180-E: Genetic, Serologic and Microbial Factors Related to Patterns of Ileal Inflammation (IPAA). Informed consent was obtained from all subjects immediately prior to the initial sample collection in compliance with our Research Ethics Board study approval. All experimental methods are compliant with the Helsinki Declaration.

For this cohort, antibiotic use was reported as ‘true’ if patients had taken antibiotics in the 30 days prior to biopsy collections. The vast majority of antibiotic use was for pouchitis, and was either metronidazole, ciprofloxacin, or a combination of both. A very small number of pouch patients (two to three) were on vancomycin instead of more standard antibiotics. Antibiotic use was also reported as ‘true’ if the patient had taken antibiotic for a non-IBD purpose in the past 30 days (for example, amoxicillin for oral surgery).

### Sample collection

Tissue biopsies were obtained from the mid-portion of the pouch and the PPI during pouchoscopy. One biopsy from each site was immediately placed into a sterile, empty freezer vial and snap frozen in liquid nitrogen for subsequent microbial analysis. Two additional biopsies from each site were placed into RNAlater (Qiagen) for host transcriptomic analysis. Study samples were stored long-term at −80°C. Two biopsies were also taken for histological analysis as per standard clinical practice at our institution. Inflammation was measured according to the objective and location-specific components from the pouchitis activity score (PAS) [59] as previously described [9,17].

### Host RNA extraction and microarray gene expression analysis

The biopsy samples were immediately suspended in RNAlater (QIAGEN) stabilizing reagent upon collection to deter RNA degradation and were stored at −80°C. Total RNA was extracted with the miRNeasy Mini Kit (Qiagen) in two batches. A NanoDrop 1000 (Thermo Fisher Scientific) and Bioanalyzer 2100 (Agilent) were used to determine RNA concentration, quality and purity. Only samples with a RNA integrity number (RIN) greater than or equal to 5.0 were considered for further analysis [60].

From samples that passed quality control, 400 ng of RNA was amplified with the Ambion WT Expression Kit (Ambion). A total of 5.5 μg of cDNA per sample were then labeled and hybridized to Human Gene 1.0 ST arrays (Affymetrix) in a Fluidics Station 450 (Affymetrix), utilizing standard protocol FS450_0007 with the GeneChip WT Terminal Labeling and Controls Kit (Affymetrix) and GeneChip Hybridization, Wash, and Stain Kit (Affymetrix). The GeneChip Scanner 3000 (Affymetrix) was used to scan the completed arrays. Summarized probe cell intensity data were generated with an Affymetrix GeneChip Command Console. Finally, probe-level summarization files were produced, and the data were background-adjusted, normalized, and log-transformed with the robust multiarray average (RMA) algorithm in Affymetrix Expression Console [61].

The empirical Bayes (EB) method described by Johnson *et al*. [62] was applied to the normalized data to correct for batch effects which may have resulted from a non-linear sample extraction and microarray processing schedule. Finally, duplicate and ambiguous Affymetrix probesets (Release 32) as well as those no longer mapping to a gene in the current human genome build (GRCh37.p5) were removed from further analysis. This filter retained 19,908 probesets from the original 33,297.

### Microbial DNA extraction and sequencing

#### Community DNA extraction

Total microbial DNA was extracted from biopsies in two batches using the DNeasy blood and tissue kit (Qiagen), with an additional bead beating step to ensure adequate cell lysis. Bead beating was performed using both 5 mm stainless steel beads to disrupt tissue (Qiagen 69989) and glass beads (Mo-Bio, Mississauga, ON, Canada) to disrupt bacterial cells, in conjunction with the FastPrep tissue homogenizer (MP Biomedicals, Santa Ana, CA, USA) set to speed 6 for 30 s. Additional enzymatic lysis was conducted through the addition of proteinase K (as per the Qiagen protocol) and incubation of samples at 95°C.

#### 16S profiling and sequencing

The 16S gene dataset consists of Illumina MiSeq sequences targeting the V4 variable region. Detailed protocols used for 16S amplification and sequencing are as previously described [63]. In brief, genomic DNA was subjected to 16S amplifications using primers designed to incorporate both the Illumina adapters and a sample barcode sequence, allowing directional sequencing that covers variable region V4 (Primers: 515 F [GTGCCAGCMGCCGCGGTAA] and 806R [GGACTACHVGGGTWTCTAAT]). PCR mixtures contained 10 μL of diluted template (1:50), 10 μL of HotMasterMix with the HotMaster Taq DNA Polymerase (5 Prime), and 5 μL of primer mix (2 μM of each primer). The cycling conditions consisted of an initial denaturation of 94°C for 3 min, followed by 30 cycles of denaturation at 94°C for 45 s, annealing at 50°C for 60 s, extension at 72°C for 5 min, and a final extension at 72°C for 10 min. Amplicons were quantified on the Caliper LabChipGX (PerkinElmer, Waltham, MA, USA), pooled in equimolar concentrations, and size selected (375–425 bp) on the Pippin Prep (Sage Sciences, Beverly, MA, USA) to reduce non-specific amplification products from host DNA. Finally, an Agilent Bioanalyzer (2100 DNA 1000 chips) (Agilent Technologies, Santa Clara, CA, USA) was used to determine the final concentration and size distribution of the library. Sequencing was performed on the Illumina MiSeq v2 platform, according to the manufacturer’s specifications, with addition of 5% PhiX, generating paired-end reads of 175 bp in length in each direction.

#### Bioinformatic processing of sequences

The overlapping paired-end reads were stitched together (approximately 97 bp overlap), size selected to reduce non-specific amplification products from host DNA (225–275 bp), and further processed in a data curation pipeline implemented in QIIME 1.5.0 as pick_reference_otus.py [64]. In brief, this pipeline picks OTUs using a reference-based method and constructs an OTU table. Taxonomy is assigned using the Greengenes predefined taxonomy map of reference sequence OTUs to taxonomy [65]. The resulting OTU tables are checked for mislabeling [66] and contamination [67], and further microbial community analysis and visualizations. A mean sequence depth of 29,914 sequences/sample was obtained, and samples with less than 3,000 filtered sequences were excluded from analysis.

### Power calculations and gene/microbial feature selection

#### Initial power calculation

Power estimation was performed by simulation of correlated variable pairs with standard normal distribution and a sample size of 196. The 90th percentile of raw *P* values of the Spearman correlation test was calculated as a function of true covariance of the variables. The number of allowable tests for 90% power and 5% type I error rate was estimated by Bonferroni correction, 0.05 divided by the 90th percentile calculated as above. The number of allowable tests increases with the assumed true covariance of the variable pair, but is approximately 100 for a true covariance of 0.35, and 10^5^ for a true covariance of 0.45 (Additional file 1A). This analysis was performed by the associated *corpower.Rnw* script.

#### Microbial feature reduction

The data were first filtered by removing OTUs without at least three counts in at least three samples. Next, OTUs were hierarchically summed at all taxonomic levels, and these counts were normalized to relative abundance. Features were then filtered again to require a mean abundance across all samples of at least 0.005, and an abundance of 0.05 in at least one sample. This left 129 features, to which we applied unsupervised (PCA) and supervised (hierarchical clustering) reduction. For PCA, a variance-stabilizing arcsine square-root transformation was applied. Next, standard Principal Component Analysis of scaled features was used to capture major axes of variation, keeping enough components to account for 50% of variance. The previously documented ‘horseshoe effect’ in Principal Component Analysis of compositional data [68] was present (Additional file 1B) but was not so extreme as to overly diminish the utility of Principal Component Analysis. Interpretation of microbial principal components was guided by a loadings plot (Figure 1B, Additional file 3A and B, Additional file 1E). PCA reduced the 129 clades to nine cPCs. For supervised feature reduction to allow pairwise comparison to host transcriptome features, we performed hierarchical clustering of clades with abundance of at least 10 to 4 in 10% of samples, 1 minus Pearson correlation dissimilarity measure, and default options for the hclust R function, then finally cutting the tree at height 0.5 and selecting the feature with smallest mean. This approach was confirmed visually to select reasonable microbial representatives (Figure 1C). This analysis was performed by the associated preparePCLfiles.Rnw script. It reduced the total number of features from 129 to 45.

### Host transcriptome feature reduction

*Supervised feature reduction:* Targeted gene selection was applied to the transcriptomic data in order to reduce its dimensionality. In a first wave of filtering, 174 genes prioritized as IBD-associated in the most recent and largest genome wide association study of the disease [4] were selected for further statistical analysis. In addition, 272 genes which were previously shown to physically interact with bacterial partners from *Bacillus anthracis*, *Francisella tularensis*, and *Yersinia pestis* based on yeast two-hybrid experiments [22] were also chosen. Preselected genes were then aggregated into 75 clusters based on their co-expression pattern using the Pearson metric and semi-supervised Ward clustering [69]. A representative gene was selected from each cluster by the k-medoids algorithm [70]. Finally, due to their importance to the pathogenesis of IBD, the following genes were manually curated and added to the existing medoids: *NOD2*, *IL23R*, *PTPN22*, *FUT2*, *NFKB1*, *MMEL1*, *IFNG*, *IL10*, *IL1RN*, *CD14*, *IL8*, *TLR1*, *TNF*, and *NOX3*.

*Unsupervised transcript reduction:* Principal component analysis of host transcriptome data was performed on all PPI and pouch samples, keeping a sufficient number of components to account for 50% of variance. The only filter applied to whole-transcriptome data for PCA was to remove transcripts with variance below the median variance of all transcripts (for example, filtering out the least-invariant two quantiles of transcripts). Interpretation of the principal component axes was assisted by inspection of the top 25 genes by magnitude of loadings, and by Enrichment Analysis using the *wilcoxGST* function of the *limma* package with ‘C2.CP.biocarta’ v3.1 mSigDB pathways [71] (Additional file 1C). This analysis was performed by the associated *PCA.Rnw* script.

### Major phenotypic associations of the microbiome and host transcriptome

A linear model was fit for each microbial clade and for each transcript separately, with respect to antibiotics (yes/no), outcome (NP, P, CDL, AP, and FAP), inflammation (0–13), and tissue location (pouch/PPI), using the *lm* R function. Nominal statistical significance of each feature was assessed by analysis of variance F-test of the fit. For the effect of tissue location, all 255 pouch and pre-pouch ileum (PPI) samples were used; for antibiotics, inflammation, outcome, and the PPI samples from each of the 196 individuals were used. The latter tests were repeated using all samples, with a random intercept for individual, using the *glmmPQL* function of the *MASS* R package. This analysis was performed for whole transcriptome data, and for all microbial clades passing the ‘3 counts in 3 samples’ filter described above, by the associated *sourcesOfVariation.Rnw* script.

### Using biplots to visualize associations between transcripts, clades, and metadata

We used the scriptBiplotTSV.R script from the Breadcrumbs software package [23] to generate a biplot showing the relationships between clades, metadata, and transcripts of interest (Figure 2). This script plots a tsv (transposed PCL) file as a biplot. The positioning of sample markers and clade text is generated by nonmetric multidimensional scaling (R Vegan package). The metadata are represented by arrows, labeled by text at the head of the arrow. Arrow coordinates are determined by the coordinates of the samples and show the central tendency of the metadata.

### Using multivariate analysis with linear modeling to model host/microbe metadata associations

MaAsLin (multivariate analysis with linear modeling) [5,28] was used to find associations between microbes, transcripts, and metadata. As many of the strongest univariate associations in this dataset (for example, chronic pouchitis and abundant *Enterococcus*) would be obviously due to either antibiotic use or inflammation, and thus of less interest than associations which were not directly attributable to either, we used a multivariate linear model to correct for antibiotic use, FAP/nonFAP outcome, and inflammation score. The model used was gene ~ clade + antibiotic + ISCORE + OutcomeFAP/nonFAP, with arcsin-square root variance stabilizing transformation of clade. Bonferroni false discovery correction was used with a threshold of q <0.25. Input files used for MaAsLin are available from [72].

### Discriminant assessment of host/microbe interactions in pouchitis outcomes

Linear discriminant analysis (LDA) was used to discriminate clinical outcome (AP, CP, NP, FAP, CDL) based on expression patterns of 75 gene medoids and 45 clades. As there were many more PPI samples (196) than pouch samples (59), to ensure all samples were equally represented, only PPI samples were used. Because antibiotic use was not uniformly distributed across outcomes, we removed all samples with recent antibiotic use for discrimination of clinical outcome. This left 55 AP samples, 18 CDL samples, five CP samples, 20 FAP samples, and 46 NP samples for LDA analysis. Discrimination models were fit with three different sets of covariates: transcripts only, clades only, and transcripts plus clades together. Model fitting and assessment of discrimination by 10-fold cross validation were performed using the R package ‘caret,’ within the script ldaprediction.Rnw from [73].

Ten-fold cross-validation was used to calculate accuracy of discrimination. For each clinical outcome and each model (transcripts only, clades only, and clades + transcripts), a ROC plot was constructed using the roc function from the pROC library, using the 10-fold cross-validated posterior probabilities from the lda function of the MASS library. Ninety-five percent confidence intervals were estimated using the ci function from the pROC package (Additional file 6).

### Data availability

16S sequence data for this project have been filtered to remove human sequences and are publicly available as Bioproject PRJNA269954; dbGaP accession number: phs000659.v1.p1 contains a subset of these data. Microarray data are available from GEO as GSE65270; GSE40292 contains a subset of these data. Metadata are available at [74].

## Additional files

Additional file 1: Figure S1. Data reduction. (A) (Top) 90th percentile of raw *P* values of Spearman correlation test, as a function of true covariance between the variables. Variables are standard normal distributed, so covariance equals Pearson product moment. (Bottom) Number of tests possible to retain 90% power and alpha equal to 0.05, using Bonferroni correction. Variables are standard normal distributed, so covariance equals Pearson product moment. (B) Principal component analysis for cPC1 and cPC2. The documented ‘horseshoe effect’ is noticeable, but not extreme. (C) Gene set enrichment analysis (GSEA) was used to detect categories for which the gPCs were enriched and assist in interpretation (see Methods). Only gPCs and gene sets with at least one significant *P* value after Bonferroni correction (q <0.1) are shown. (D) The top 25 loading values for each clade principal component. The blue/orange scale bar corresponds to a decrease or increase in the relative abundance of the clade in the principal component. Additional file 2: Figure S2. The transcriptome and microbiome in paired samples. The Pearson correlation was calculated for host transcripts in all paired pouch-PPI samples, and the Bray-Curtis distance was calculated for all microbiome samples. Ordinations were calculated for Bray-Curtis and for (1-Pearson correlation). Paired samples are connected with a line on ordinations. Plots show the difference between samples between locations for genes (top) and for microbes (bottom). Additional file 3
**Supplementary data tables.** A: The top 25 loadings for each clade principal component (cPC). B: The top 25 loadings for each gene (host transcript) principal component (gPC). C: The list of 75 gene medoids, each of which represents a cluster of genes with a similar expression profile. D: List of *P* values of differential expression in pouch for all metadata for all clades. E: List of *P* values of differential expression in pre-pouch ileum for all metadata for all clades. F: List of *P* values of differential expression in pouch for all metadata for all genes. G: List of *P* values of differential expression in pre-pouch ileum for all metadata for all genes. H: List of *P* values of differential expression in all samples for all metadata for all genes, calculated using random intercept of individual. I: List of *P* values of differential expression in all samples for all metadata for all clades, calculated using random intercept of individual. Additional file 4: Figure S3. GOrilla analysis. GOrilla was used to measure for functional enrichment between genes differentially expressed in pouch and pre-pouch ileum (Additional file 3). There was a major difference in transporter expression between the two sites. Additional file 5
**Figure S4.** Data stratification. (A) cPC1, cPC3, cPC6, cPC8, gPC8, and gPC9 were the principal components that significantly associated with one another in multivariate linear analysis. This figure shows the expression of each of these components in PPI samples when stratified by antibiotic use and by clinical outcome. (B) This figure shows the distribution of antibiotic use in the cohort, stratified by sample type (pouch vs. PPI) and clinical outcome. (C) The distribution of Enterococcacaeae in samples, stratified by clinical outcome and antibiotic use. It is abundant almost exclusively in chronic pouchitis patients with recent antibiotic use. (D) gPC9, plotted relative to patient histological inflammation score. (E) *Escherichia* abundance, plotted relative to gPC9. Additional file 6: Figure S5. Linear discriminant analysis for discrimination of clinical outcome. (A) Summary of LDA prediction for samples without antibiotics. Top: Areas under the curve for LDA discrimination models. A single model was fit with 5-level response. Ten-fold cross-validated class probabilities for each level (AP, CDL, CP, NP, FAP) were used to construct ROC plots for that outcome. Ninety-five percent confidence intervals were estimated using the ci function from the pROC package. Bottom: Individual ROC plots for each possible outcome, using genes only, clades only, and genes + clades. For each model, the ROC plot was constructed using the roc function from the pROC library, from 10-fold cross-validated posterior probabilities from the lda function of the MASS library. (B) Summary of LDA prediction using all samples (with and without antibiotics). These were calculated as described in (A). (C) LDA score scatterplots for the phenotypes show which LDAs discriminate for which phenotypes. Only the scatterplots for antibiotic-free samples are shown. Scatterplots for genes (left) and for clades (right) are shown. Scatterplots are colored for visualization. (D) Linear discriminant loadings plots show which genes and microbes are most elevated or decreased in LDs 1 to 4 (and are thus most discriminant).

## References

[1] Landy J, Al-Hassi HO, McLaughlin SD, Knight SC, Ciclitira PJ, Nicholls RJ (2012). Etiology of pouchitis. Inflamm Bowel Dis.

[2] Tyler AD, Milgrom R, Stempak JM, Xu W, Brumell JH, Muise AM (2013). The NOD2insC polymorphism is associated with worse outcome following ileal pouch-anal anastomosis for ulcerative colitis. Gut.

[3] McLaughlin SD, Clark SK, Tekkis PP, Nicholls RJ, Ciclitira PJ (2010). The bacterial pathogenesis and treatment of pouchitis. Therap Adv Gastroenterol.

[4] Jostins L, Ripke S, Weersma RK, Duerr RH, McGovern DP, Hui KY (2012). Host-microbe interactions have shaped the genetic architecture of inflammatory bowel disease. Nature.

[5] Morgan XC, Tickle TL, Sokol H, Gevers D, Devaney KL, Ward DV (2012). Dysfunction of the intestinal microbiome in inflammatory bowel disease and treatment. Genome Biol.

[6] Baumgart M, Dogan B, Rishniw M, Weitzman G, Bosworth B, Yantiss R (2007). Culture independent analysis of ileal mucosa reveals a selective increase in invasive Escherichia coli of novel phylogeny relative to depletion of Clostridiales in Crohn's disease involving the ileum. Isme J.

[7] Joossens M, Huys G, Cnockaert M, De Preter V, Verbeke K, Rutgeerts P (2011). Dysbiosis of the faecal microbiota in patients with Crohn’s disease and their unaffected relatives. Gut.

[8] Ott SJ, Musfeldt M, Wenderoth DF, Hampe J, Brant O, Folsch UR (2004). Reduction in diversity of the colonic mucosa associated bacterial microflora in patients with active inflammatory bowel disease. Gut.

[9] Tyler AD, Knox N, Kabakchiev B, Milgrom R, Kirsch R, Cohen Z (2013). Characterization of the gut-associated microbiome in inflammatory pouch complications following ileal pouch-anal anastomosis. PLoS One.

[10] Khan KJ, Ullman TA, Ford AC, Abreu MT, Abadir A, Marshall JK (2011). Antibiotic therapy in inflammatory bowel disease: a systematic review and meta-analysis. Am J Gastroenterol.

[11] Wang SL, Wang ZR, Yang CQ (2012). Meta-analysis of broad-spectrum antibiotic therapy in patients with active inflammatory bowel disease. Exp Ther Med.

[12] Wu H, Shen B (2009). Pouchitis: lessons for inflammatory bowel disease. Curr Opin Gastroenterol.

[13] de Silva HJ, Millard PR, Kettlewell M, Mortensen NJ, Prince C, Jewell DP (1991). Mucosal characteristics of pelvic ileal pouches. Gut.

[14] Young VB, Raffals LH, Huse SM, Vital M, Dai D, Schloss PD (2013). Multiphasic analysis of the temporal development of the distal gut microbiota in patients following ileal pouch anal anastomosis. Microbiome.

[15] McLaughlin SD, Walker AW, Churcher C, Clark SK, Tekkis PP, Johnson MW (2010). The bacteriology of pouchitis: a molecular phylogenetic analysis using 16S rRNA gene cloning and sequencing. Ann Surg.

[16] Zella GC, Hait EJ, Glavan T, Gevers D, Ward DV, Kitts CL (2011). Distinct microbiome in pouchitis compared to healthy pouches in ulcerative colitis and familial adenomatous polyposis. Inflamm Bowel Dis.

[17] Kabakchiev B, Tyler A, Stempak JM, Milgrom R, Silverberg MS (2014). Downregulation of expression of xenobiotic efflux genes is associated with pelvic pouch inflammation in ulcerative colitis. Inflamm Bowel Dis.

[18] Ben-Shachar S, Yanai H, Baram L, Elad H, Meirovithz E, Ofer A (2013). Gene expression profiles of ileal inflammatory bowel disease correlate with disease phenotype and advance understanding of its immunopathogenesis. Inflamm Bowel Dis.

[19] Ringner M (2008). What is principal component analysis?. Nat Biotech.

[20] Alter O, Brown PO, Botstein D (2000). Singular value decomposition for genome-wide expression data processing and modeling. Proc Natl Acad Sci U S A.

[21] Biswas S, Storey JD, Akey JM (2008). Mapping gene expression quantitative trait loci by singular value decomposition and independent component analysis. BMC Bioinformatics.

[22] Dyer MD, Neff C, Dufford M, Rivera CG, Shattuck D, Bassaganya-Riera J (2010). The human-bacterial pathogen protein interaction networks of Bacillus anthracis, Francisella tularensis, and Yersinia pestis. PLoS One.

[23] Breadcrumbs. [http://huttenhower.sph.harvard.edu/biobakery/breadcrumbs].

[24] Nagy E, Foldes J (1991). Inactivation of metronidazole by Enterococcus faecalis. J Antimicrob Chemother.

[25] Perry JD, Ford M, Gould FK (1994). Susceptibility of enterococci to ciprofloxacin. J Antimicrob Chemother.

[26] Eden E, Navon R, Steinfeld I, Lipson D, Yakhini Z (2009). GOrilla: a tool for discovery and visualization of enriched GO terms in ranked gene lists. BMC Bioinformatics.

[27] Segata N, Izard J, Waldron L, Gevers D, Miropolsky L, Garrett WS (2011). Metagenomic biomarker discovery and explanation. Genome Biol.

[28] MaAsLiN. [http://huttenhower.sph.harvard.edu/maaslin].

[29] Everard A, Belzer C, Geurts L, Ouwerkerk JP, Druart C, Bindels LB (2013). Cross-talk between Akkermansia muciniphila and intestinal epithelium controls diet-induced obesity. Proc Natl Acad Sci U S A.

[30] Williams BL, Hornig M, Parekh T, Lipkin WI (2012). Application of novel PCR-based methods for detection, quantitation, and phylogenetic characterization of Sutterella species in intestinal biopsy samples from children with autism and gastrointestinal disturbances. mBio.

[31] Wang L, Christophersen CT, Sorich MJ, Gerber JP, Angley MT, Conlon MA (2013). Increased abundance of Sutterella spp. and Ruminococcus torques in feces of children with autism spectrum disorder. Mol Autism.

[32] Gevers D, Kugathasan S, Denson LA, Vazquez-Baeza Y, Van Treuren W, Ren B (2014). The treatment-naive microbiome in new-onset Crohn’s disease. Cell Host Microbe.

[33] Human Microbiome Project C (2012). Structure, function and diversity of the healthy human microbiome. Nature.

[34] Qin J, Li R, Raes J, Arumugam M, Burgdorf KS, Manichanh C (2010). A human gut microbial gene catalogue established by metagenomic sequencing. Nature.

[35] Yasuda K, Oh K, Ren B, Tickle TL, Franzosa EA, Wachtman LM (2015). Biogeography of the intestinal mucosal and lumenal microbiome in the rhesus macaque. Cell Host Microbe.

[36] Sharon I, Morowitz MJ, Thomas BC, Costello EK, Relman DA, Banfield JF (2013). Time series community genomics analysis reveals rapid shifts in bacterial species, strains, and phage during infant gut colonization. Genome Res.

[37] Koenig JE, Spor A, Scalfone N, Fricker AD, Stombaugh J, Knight R (2011). Succession of microbial consortia in the developing infant gut microbiome. Proc Natl Acad Sci U S A.

[38] Makino H, Kushiro A, Ishikawa E, Kubota H, Gawad A, Sakai T (2013). Mother-to-infant transmission of intestinal bifidobacterial strains has an impact on the early development of vaginally delivered infant's microbiota. PLoS One.

[39] Kostic AD, Gevers D, Siljander H, Vatanen T, Hyotylainen T, Hamalainen AM (2015). The dynamics of the human infant gut microbiome in development and in progression toward type 1 diabetes. Cell Host Microbe.

[40] David LA, Maurice CF, Carmody RN, Gootenberg DB, Button JE, Wolfe BE (2014). Diet rapidly and reproducibly alters the human gut microbiome. Nature.

[41] Lofmark S, Edlund C, Nord CE (2010). Metronidazole is still the drug of choice for treatment of anaerobic infections. Clin Infect Dis.

[42] Sheng WS, Hsueh PR, Hung CC, Teng LJ, Chen YC, Luh KT (2001). Clinical features of patients with invasive Eikenella corrodens infections and microbiological characteristics of the causative isolates. Eur J Clin Microbiol Infect Dis.

[43] Lubbe MM, Stanley K, Chalkley LJ (1999). Prevalence of nim genes in anaerobic/facultative anaerobic bacteria isolated in South Africa. FEMS Microbiol Lett.

[44] de Been M, van Schaik W, Cheng L, Corander J, Willems RJ (2013). Recent recombination events in the core genome are associated with adaptive evolution in Enterococcus faecium. Genome Biol Evol.

[45] Rams TE, Feik D, Mortensen JE, Degener JE, van Winkelhoff AJ (2013). Antibiotic susceptibility of periodontal Enterococcus faecalis. J Periodontol.

[46] Lucas GM, Lechtzin N, Puryear DW, Yau LL, Flexner CW, Moore RD (1998). Vancomycin-resistant and vancomycin-susceptible enterococcal bacteremia: comparison of clinical features and outcomes. Clin Infect Dis.

[47] Rafii F, Wynne R, Heinze TM, Paine DD (2003). Mechanism of metronidazole-resistance by isolates of nitroreductase-producing Enterococcus gallinarum and Enterococcus casseliflavus from the human intestinal tract. FEMS Microbiol Lett.

[48] Jia W, Li G, Wang W (2014). Prevalence and antimicrobial resistance of Enterococcus species: a hospital-based study in China. Int J Environ Res Public Health.

[49] Sadowy E, Sienko A, Gawryszewska I, Bojarska A, Malinowska K, Hryniewicz W (2013). High abundance and diversity of antimicrobial resistance determinants among early vancomycin-resistant Enterococcus faecium in Poland. Eur J Clin Microbiol Infect Dis.

[50] Sreeja S, Babu PRS, Prathab AG (2012). The prevalence and the characterization of the enterococcus species from various clinical samples in a tertiary care hospital. J Clin Diagn Res.

[51] Gutierrez Martin CB, Rodriguez Ferri EF (1993). In vitro susceptibility of Pasteurella multocida subspecies multocida strains isolated from swine to 42 antimicrobial agents. Zentralbl Bakteriol.

[52] Elliott TR, Hudspith BN, Wu G, Cooley M, Parkes G, Quinones B (2013). Quantification and characterization of mucosa-associated and intracellular Escherichia coli in inflammatory bowel disease. Inflamm Bowel Dis.

[53] Winter SE, Winter MG, Xavier MN, Thiennimitr P, Poon V, Keestra AM (2013). Host-derived nitrate boosts growth of E. coli in the inflamed gut. Science.

[54] Kang CS, Ban M, Choi EJ, Moon HG, Jeon JS, Kim DK (2013). Extracellular vesicles derived from gut microbiota, especially Akkermansia muciniphila, protect the progression of dextran sulfate sodium-induced colitis. PLoS One.

[55] Ganesh BP, Klopfleisch R, Loh G, Blaut M (2013). Commensal Akkermansia muciniphila exacerbates gut inflammation in Salmonella Typhimurium-infected gnotobiotic mice. PLoS One.

[56] Race AM, Steven RT, Palmer AD, Styles IB, Bunch J (2013). Memory efficient principal component analysis for the dimensionality reduction of large mass spectrometry imaging data sets. Anal Chem.

[57] Engreitz JM, Daigle BJ, Marshall JJ, Altman RB (2010). Independent component analysis: mining microarray data for fundamental human gene expression modules. J Biomed Inform.

[58] Korkeila EA, Sundstrom J, Pyrhonen S, Syrjanen K (2011). Carbonic anhydrase IX, hypoxia-inducible factor-1alpha, ezrin and glucose transporter-1 as predictors of disease outcome in rectal cancer: multivariate Cox survival models following data reduction by principal component analysis of the clinicopathological predictors. Anticancer Res.

[59] Heuschen UA, Autschbach F, Allemeyer EH, Zollinger AM, Heuschen G, Uehlein T (2001). Long-term follow-up after ileoanal pouch procedure: algorithm for diagnosis, classification, and management of pouchitis. Dis Colon Rectum.

[60] Schroeder A, Mueller O, Stocker S, Salowsky R, Leiber M, Gassmann M (2006). The RIN: an RNA integrity number for assigning integrity values to RNA measurements. BMC Mol Biol.

[61] Irizarry RA, Hobbs B, Collin F, Beazer-Barclay YD, Antonellis KJ, Scherf U (2003). Exploration, normalization, and summaries of high density oligonucleotide array probe level data. Biostatistics.

[62] Johnson WE, Li C, Rabinovic A (2007). Adjusting batch effects in microarray expression data using empirical Bayes methods. Biostatistics.

[63] Caporaso JG, Lauber CL, Walters WA, Berg-Lyons D, Huntley J, Fierer N (2012). Ultra-high-throughput microbial community analysis on the Illumina HiSeq and MiSeq platforms. ISME J.

[64] Caporaso JG, Kuczynski J, Stombaugh J, Bittinger K, Bushman FD, Costello EK (2010). QIIME allows analysis of high-throughput community sequencing data. Nat Methods.

[65] McDonald D, Price MN, Goodrich J, Nawrocki EP, DeSantis TZ, Probst A (2012). An improved Greengenes taxonomy with explicit ranks for ecological and evolutionary analyses of bacteria and archaea. ISME J.

[66] Knights D, Kuczynski J, Koren O, Ley RE, Field D, Knight R (2011). Supervised classification of microbiota mitigates mislabeling errors. ISME J.

[67] Knights D, Kuczynski J, Charlson ES, Zaneveld J, Mozer MC, Collman RG (2011). Bayesian community-wide culture-independent microbial source tracking. Nat Methods.

[68] Legendre P, Gallagher E (2001). Ecologically meaningful transformations for ordination of species data. Oecologia.

[69] Ward JH (1963). Hierarchical grouping to optimize an objective function. J Am Stat Assoc.

[70] Hastie T, Tibshirani R, Friedman J (2009). The elements of statistical learning: data mining, inference and prediction.

[71] Gene Set Enrichment Analysis. [http://www.broadinstitute.org/gsea]

[72] Pouchitis. [https://bitbucket.org/biobakery/pouchitis-public/]

[73] Pouchitis Source. [https://bitbucket.org/biobakery/pouchitis-public/src]

[74] Pouchitis2015. [http://huttenhower.sph.harvard.edu/pouchitis2015]

