# Supplementary figures and images for: Associations between host gene expression, the mucosal microbiome, and clinical outcome in the pelvic pouch of patients with inflammatory bowel disease
Source: Genome Biol. 2015 Apr 8;16(1):67. doi: 10.1186/s13059-015-0637-x (PMC4414286; doi:10.1186/s13059-015-0637-x)

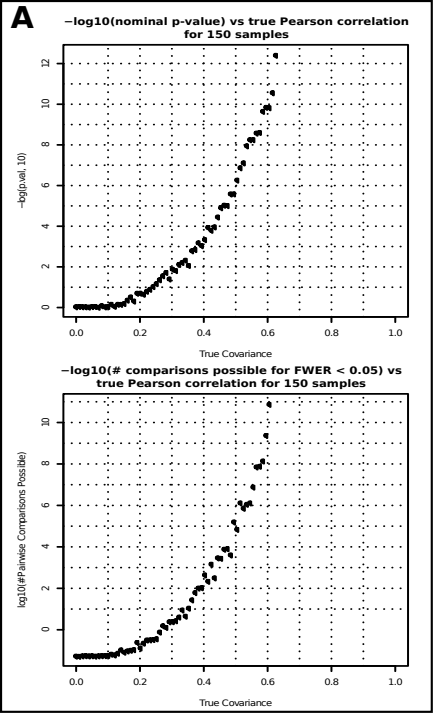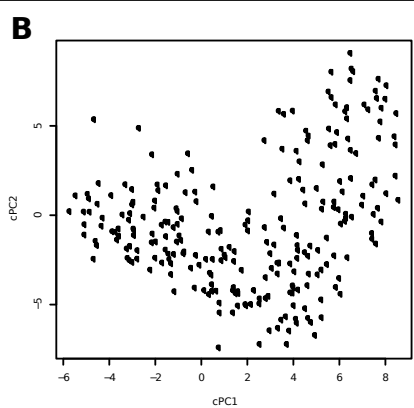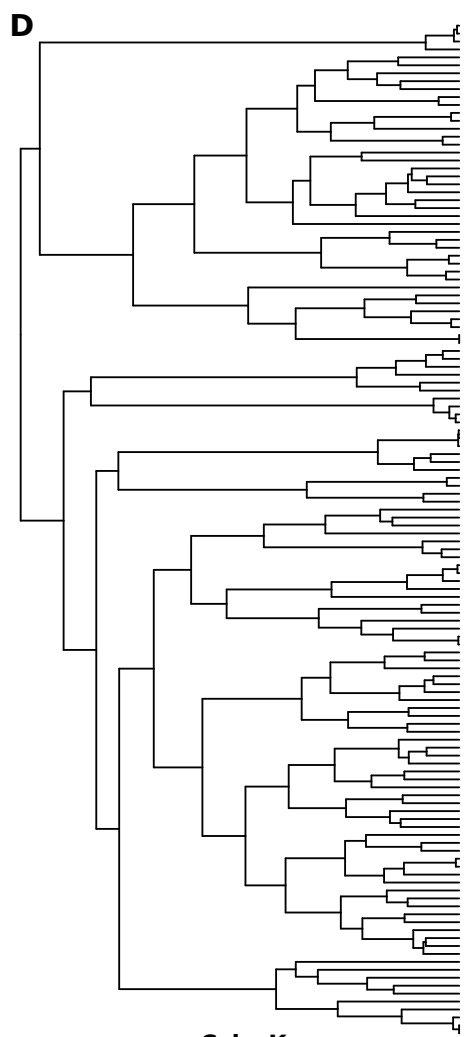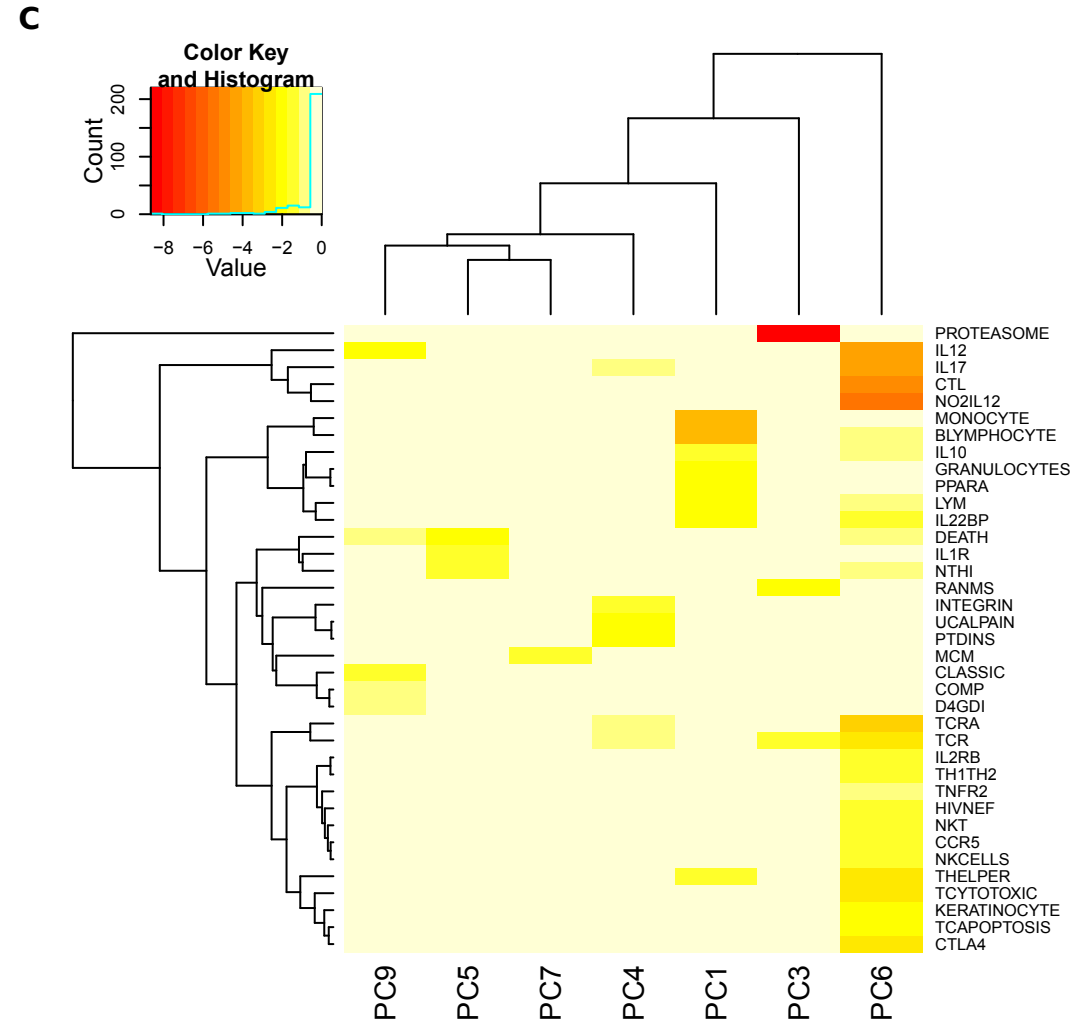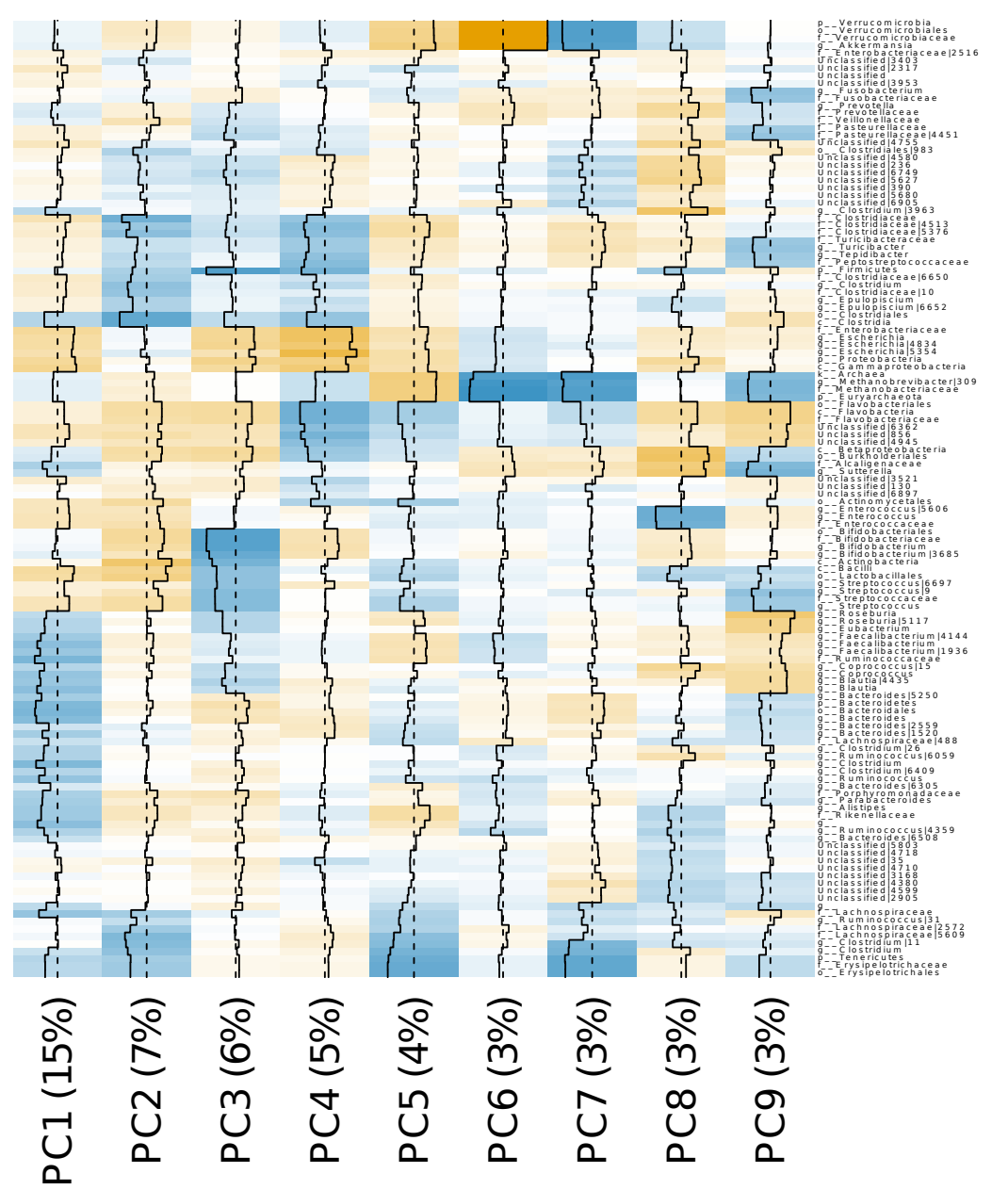

Supplement: Additional file 1: Figure S1. — Data reduction. (A) (Top) 90th percentile of raw P values of Spearman correlation test, as a function of true covariance between the variables. Variables are standard normal distributed, so covariance equals Pearson product moment. (Bottom) Number of tests possible to retain 90% power and alpha equal to 0.05, using Bonferroni correction. Variables are standard normal distributed, so covariance equals Pearson product moment. (B) Principal component analysis for cPC1 and cPC2. The documented ‘horseshoe effect’ is noticeable, but not extreme. (C) Gene set enrichment analysis (GSEA) was used to detect categories for which the gPCs were enriched and assist in interpretation (see Methods). Only gPCs and gene sets with at least one significant P value after Bonferroni correction (q <0.1) are shown. (D) The top 25 loading values for each clade principal component. The blue/orange scale bar corresponds to a decrease or increase in the relative abundance of the clade in the principal component. [file 13059_2015_637_MOESM1_ESM.pdf]

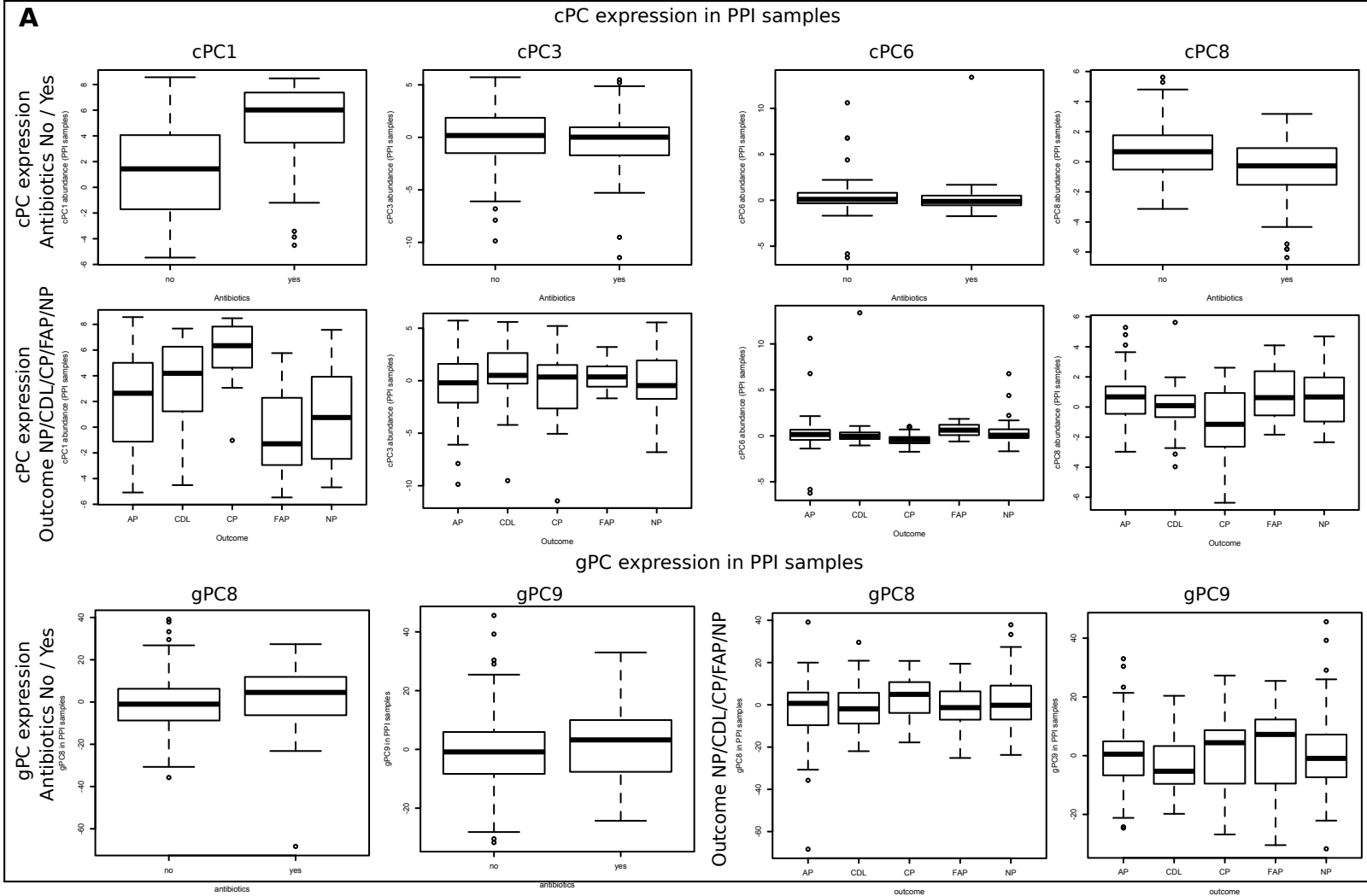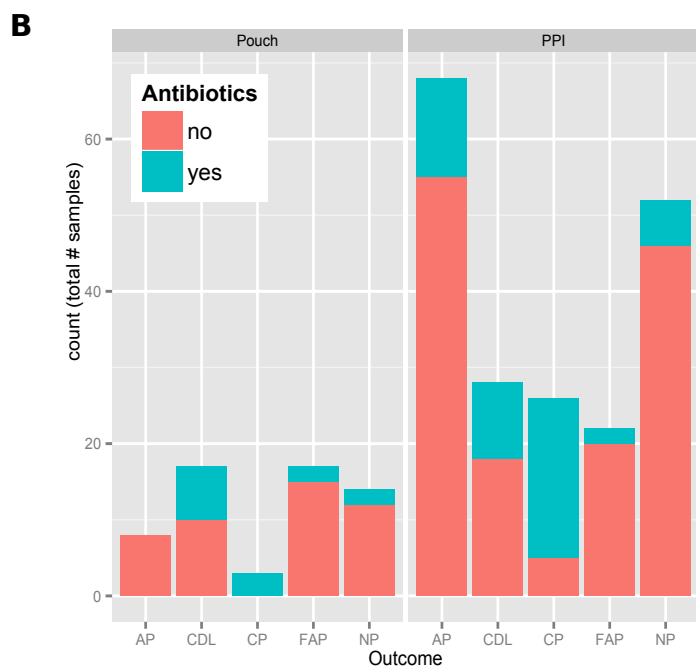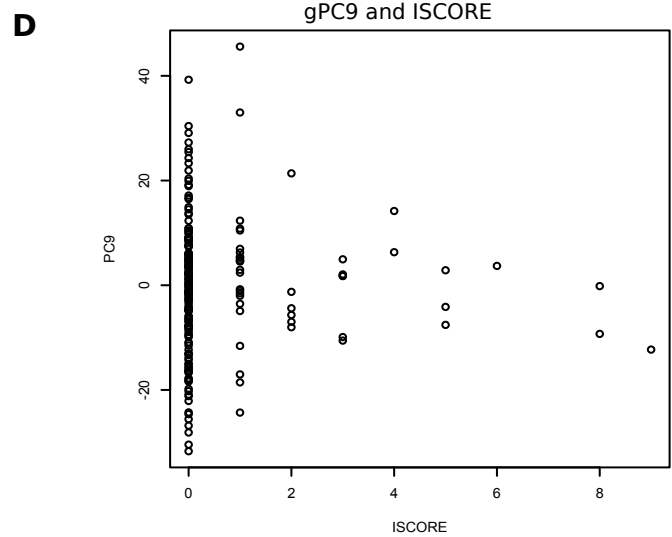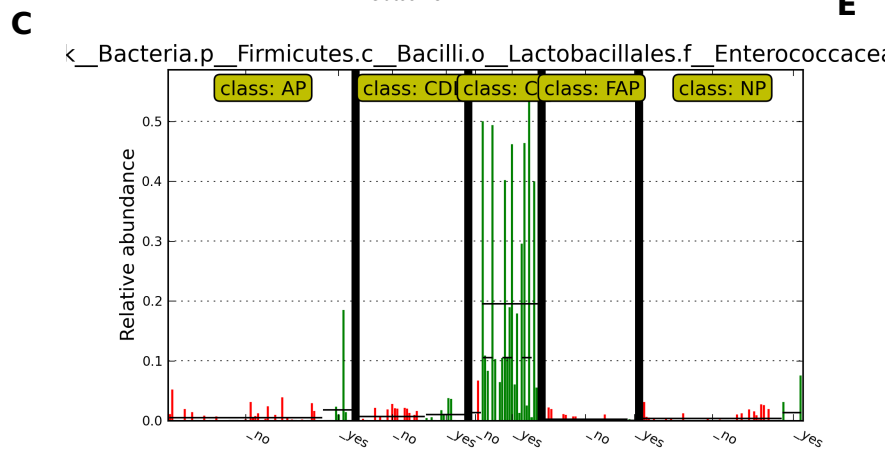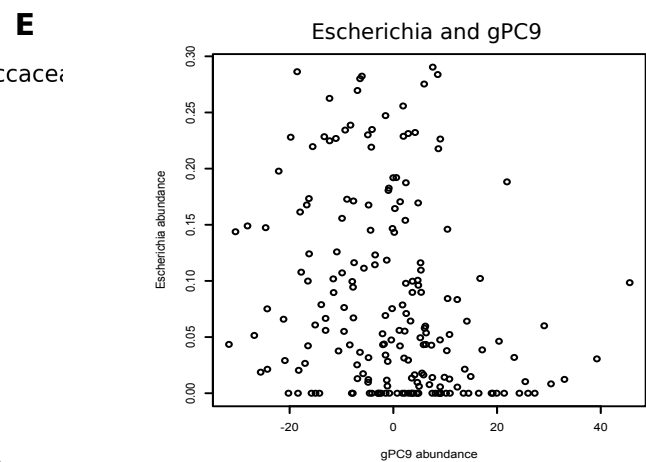

Supplement: Additional file 5 — Figure S4. Data stratification. (A) cPC1, cPC3, cPC6, cPC8, gPC8, and gPC9 were the principal components that significantly associated with one another in multivariate linear analysis. This figure shows the expression of each of these components in PPI samples when stratified by antibiotic use and by clinical outcome. (B) This figure shows the distribution of antibiotic use in the cohort, stratified by sample type (pouch vs. PPI) and clinical outcome. (C) The distribution of Enterococcacaeae in samples, stratified by clinical outcome and antibiotic use. It is abundant almost exclusively in chronic pouchitis patients with recent antibiotic use. (D) gPC9, plotted relative to patient histological inflammation score. (E) Escherichia abundance, plotted relative to gPC9. [file 13059_2015_637_MOESM5_ESM.pdf]
