# Supplementary material for: Associations between host gene expression, the mucosal microbiome, and clinical outcome in the pelvic pouch of patients with inflammatory bowel disease
Source: Genome Biol. 2015 Apr 8;16(1):67. doi: 10.1186/s13059-015-0637-x (PMC4414286; doi:10.1186/s13059-015-0637-x)
Supplement: Additional file 2: Figure S2. — The transcriptome and microbiome in paired samples. The Pearson correlation was calculated for host transcripts in all paired pouch-PPI samples, and the Bray-Curtis distance was calculated for all microbiome samples. Ordinations were calculated for Bray-Curtis and for (1-Pearson correlation). Paired samples are connected with a line on ordinations. Plots show the difference between samples between locations for genes (top) and for microbes (bottom). [file 13059_2015_637_MOESM2_ESM.pdf]

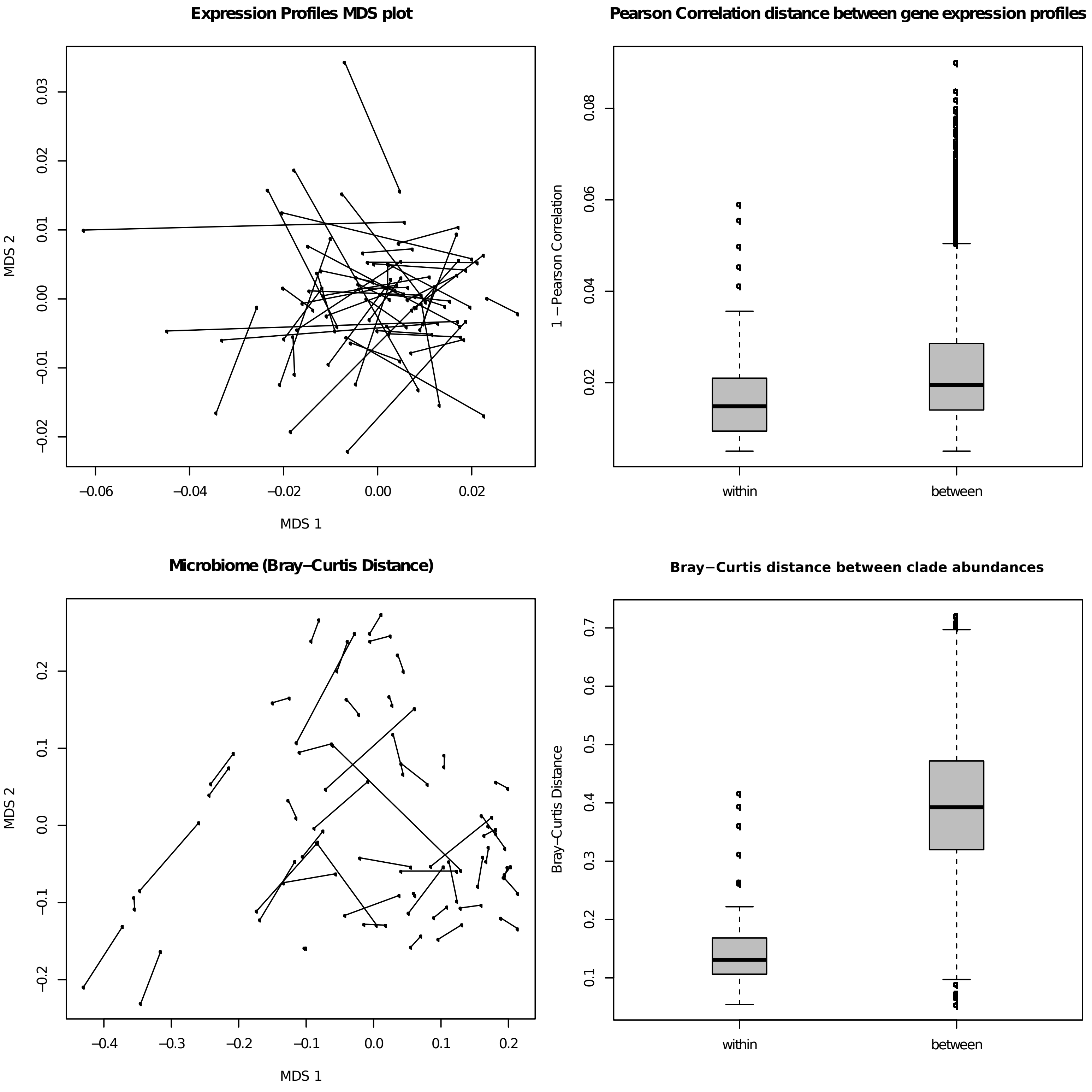

Expression Profiles MDS plot
MDS 1
MDS 2
−0.06
−0.04
−0.02
0.00
0.02
−0.02
−0.01
0.00
0.01
0.02
0.03
Pearson Correlation distance between gene expression profiles
1 − Pearson Correlation
0.02
0.04
0.06
0.08
within
between
Microbiome (Bray−Curtis Distance)
MDS 1
MDS 2
−0.4
−0.3
−0.2
−0.1
0.0
0.1
0.2
−0.2
−0.1
0.0
0.1
0.2
Bray−Curtis distance between clade abundances
Bray−Curtis Distance
0.1
0.2
0.3
0.4
0.5
0.6
0.7
within
between
