# Supplementary material for: Associations between host gene expression, the mucosal microbiome, and clinical outcome in the pelvic pouch of patients with inflammatory bowel disease
Source: Genome Biol. 2015 Apr 8;16(1):67. doi: 10.1186/s13059-015-0637-x (PMC4414286; doi:10.1186/s13059-015-0637-x)
Supplement: Additional file 4: Figure S3. — GOrilla analysis. GOrilla was used to measure for functional enrichment between genes differentially expressed in pouch and pre-pouch ileum (Additional file 3). There was a major difference in transporter expression between the two sites. [file 13059_2015_637_MOESM4_ESM.pdf]

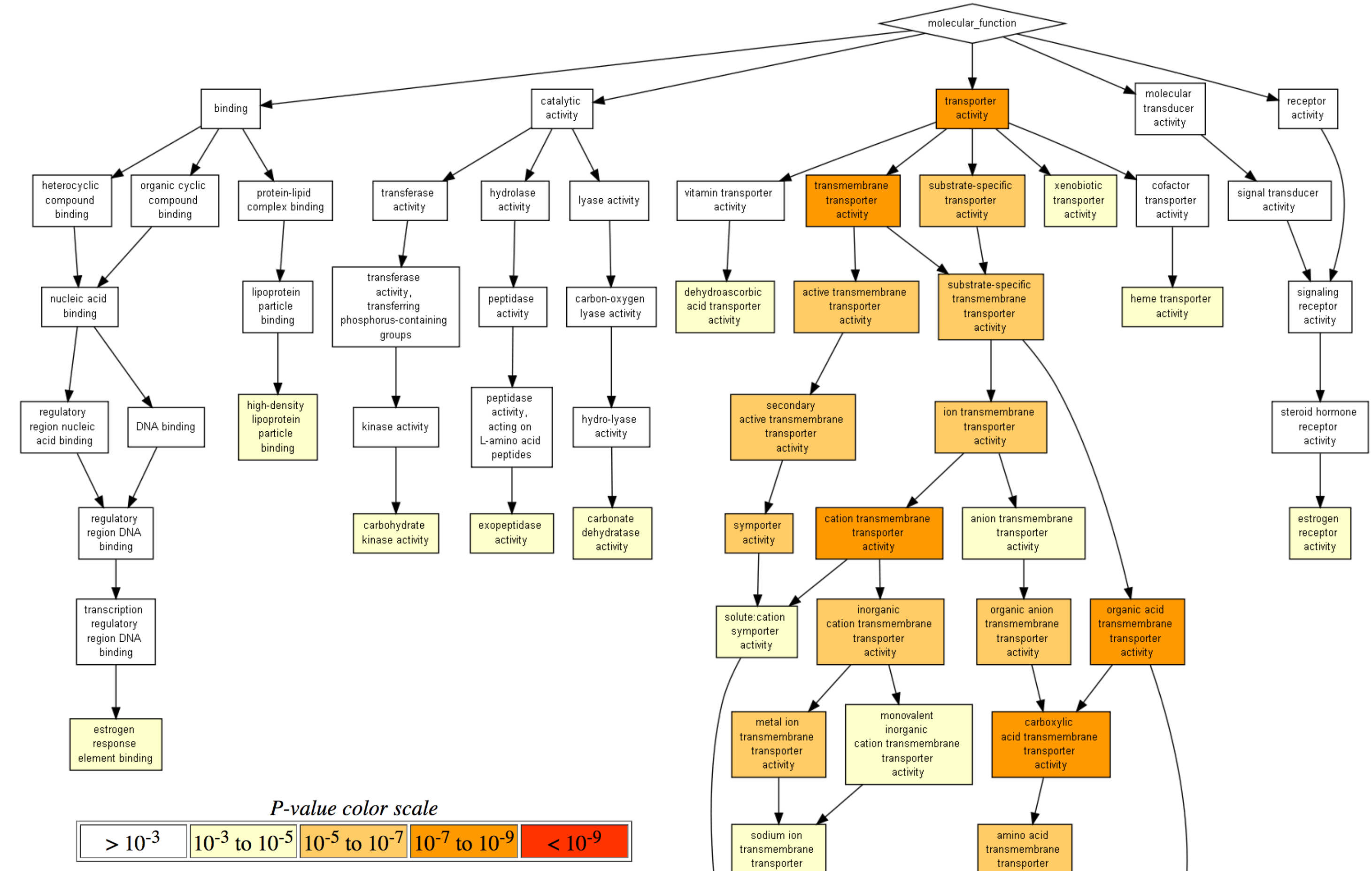

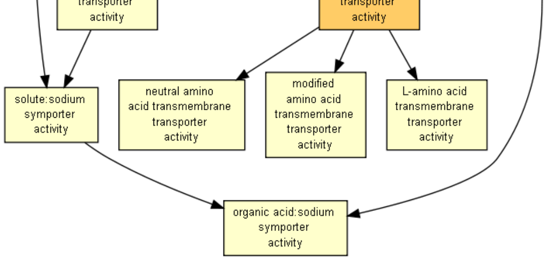

GO term	Description	P-value	FDR q-value	Enrichment (N, B, n, b)	Genes
GO:0005215	transporter activity	1.54E-9	4.98E-6	2.47 (8054,546,341,57)	[+] Show genes
GO:0022857	transmembrane transporter activity	1.61E-8	2.59E-5	1.85 (8054,416,975,93)	[+] Show genes
GO:0046943	carboxylic acid transmembrane transporter activity	1.63E-8	1.75E-5	4.01 (8054,53,872,23)	[+] Show genes
GO:0008324	cation transmembrane transporter activity	2.06E-8	1.66E-5	3.20 (8054,244,340,33)	[+] Show genes
GO:0005342	organic acid transmembrane transporter activity	4.1E-8	2.65E-5	3.86 (8054,55,872,23)	[+] Show genes
GO:0008514	organic anion transmembrane transporter activity	1.17E-7	6.32E-5	3.18 (8054,74,957,28)	[+] Show genes
GO:0022891	substrate-specific transmembrane transporter activity	2.03E-7	9.37E-5	1.89 (8054,372,884,77)	[+] Show genes
GO:0022804	active transmembrane transporter activity	3.15E-7	1.27E-4	2.37 (8054,154,925,42)	[+] Show genes
GO:0022892	substrate-specific transporter activity	5.04E-7	1.81E-4	2.40 (8054,434,340,44)	[+] Show genes
GO:0015075	ion transmembrane transporter activity	7.85E-7	2.53E-4	1.89 (8054,338,884,70)	[+] Show genes
GO:0046873	metal ion transmembrane transporter activity	1.04E-6	3.05E-4	3.06 (8054,165,430,27)	[+] Show genes
GO:0015291	secondary active transmembrane transporter activity	1.29E-6	3.46E-4	2.73 (8054,99,923,31)	[+] Show genes
GO:0015293	symporter activity	2.4E-6	5.96E-4	2.95 (8054,77,923,26)	[+] Show genes
GO:0022890	inorganic cation transmembrane transporter activity	2.47E-6	5.7E-4	2.83 (8054,186,444,29)	[+] Show genes
GO:0015171	amino acid transmembrane transporter activity	2.78E-6	6E-4	4.10 (8054,36,872,16)	[+] Show genes
GO:0015081	sodium ion transmembrane transporter activity	6.04E-4	6.5E-2	3.75 (8054,60,430,12)	[+] Show genes
GO:0015179	L-amino acid transmembrane transporter activity	6.73E-4	7.02E-2	3.89 (8054,25,829,10)	[+] Show genes
GO:0005343	organic acid:sodium symporter activity	8.1E-4	8.17E-2	5.08 (8054,14,793,7)	[+] Show genes
GO:0072349	modified amino acid transmembrane transporter activity	8.97E-4	8.78E-2	6.60 (8054,7,872,5)	[+] Show genes
GO:0015077	monovalent inorganic cation transmembrane transporter activity	2.25E-5	4.55E-3	3.12 (8054,128,444,22)	[+] Show genes
GO:0015370	solute:sodium symporter activity	2.62E-5	4.98E-3	4.51 (8054,27,793,12)	[+] Show genes
GO:0015294	solute:cation symporter activity	3.58E-5	6.42E-3	6.16 (8054,49,267,10)	[+] Show genes
GO:0008509	anion transmembrane transporter activity	5.01E-5	8.52E-3	2.24 (8054,128,957,34)	[+] Show genes
GO:0034056	estrogen response element binding	1.05E-4	1.69E-2	153.41 (8054,3,35,2)	[+] Show genes
GO:0004089	carbonate dehydratase activity	1.77E-4	2.73E-2	7.68 (8054,10,629,6)	[+] Show genes
GO:0042910	xenobiotic transporter activity	2.15E-4	3.15E-2	20.76 (8054,3,388,3)	[+] Show genes
GO:0019200	carbohydrate kinase activity	2.49E-4	3.5E-2	6.22 (8054,14,647,7)	[+] Show genes
GO:0015175	neutral amino acid transmembrane transporter activity	2.54E-4	3.42E-2	5.25 (8054,16,767,8)	[+] Show genes
GO:0033300	dehydroascorbic acid transporter activity	2.8E-4	3.62E-2	18.95 (8054,3,425,3)	[+] Show genes
GO:0008035	high-density lipoprotein particle binding	2.99E-4	3.72E-2	24.86 (8054,4,243,3)	[+] Show genes
GO:0030284	estrogen receptor activity	3.12E-4	3.74E-2	115.06 (8054,4,35,2)	[+] Show genes
GO:0008238	exopeptidase activity	3.53E-4	4.08E-2	4.25 (8054,54,386,11)	[+] Show genes
GO:0015232	heme transporter activity	5.22E-4	5.82E-2	25.17 (8054,6,160,3)	[+] Show genes
