# Supplementary material for: Associations between host gene expression, the mucosal microbiome, and clinical outcome in the pelvic pouch of patients with inflammatory bowel disease
Source: Genome Biol. 2015 Apr 8;16(1):67. doi: 10.1186/s13059-015-0637-x (PMC4414286; doi:10.1186/s13059-015-0637-x)
Supplement: Additional file 6: Figure S5. — Linear discriminant analysis for discrimination of clinical outcome. (A) Summary of LDA prediction for samples without antibiotics. Top: Areas under the curve for LDA discrimination models. A single model was fit with 5-level response. Ten-fold cross-validated class probabilities for each level (AP, CDL, CP, NP, FAP) were used to construct ROC plots for that outcome. Ninety-five percent confidence intervals were estimated using the ci function from the pROC package. Bottom: Individual ROC plots for each possible outcome, using genes only, clades only, and genes + clades. For each model, the ROC plot was constructed using the roc function from the pROC library, from 10-fold cross-validated posterior probabilities from the lda function of the MASS library. (B) Summary of LDA prediction using all samples (with and without antibiotics). These were calculated as described in (A). (C) LDA score scatterplots for the phenotypes show which LDAs discriminate for which phenotypes. Only the scatterplots for antibiotic-free samples are shown. Scatterplots for genes (left) and for clades (right) are shown. Scatterplots are colored for visualization. (D) Linear discriminant loadings plots show which genes and microbes are most elevated or decreased in LDs 1 to 4 (and are thus most discriminant). [file 13059_2015_637_MOESM6_ESM.pdf]

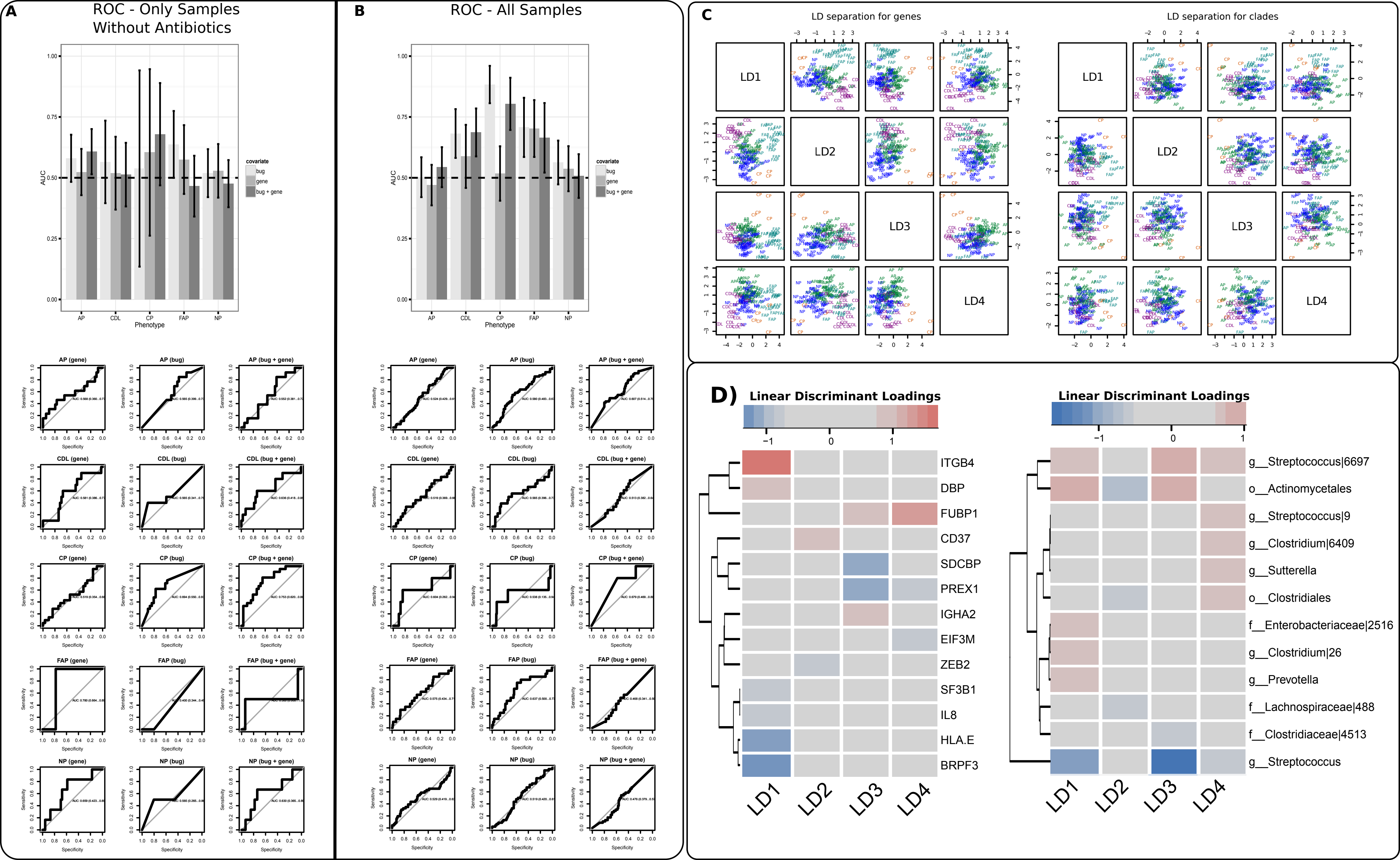

A
ROC - Only Samples Without Antibiotics
B
ROC - All Samples
AUC
Phenotype
AP
CDL
CP
FAP
NP
covariate
bug
gene
bug + gene
AP (gene)
AP (bug)
AP (bug + gene)
CDL (gene)
CDL (bug)
CDL (bug + gene)
CP (gene)
CP (bug)
CP (bug + gene)
FAP (gene)
FAP (bug)
FAP (bug + gene)
NP (gene)
NP (bug)
NP (bug + gene)
Sensitivity
Specificity
C
LD separation for genes
LD separation for clades
LD1
LD2
LD3
LD4
D)
Linear Discriminant Loadings
Linear Discriminant Loadings
ITGB4
DBP
FUBP1
CD37
SDCBP
PREX1
IGHA2
EIF3M
ZEB2
SF3B1
IL8
HLA.E
BRPF3
g__Streptococcus|6697
o__Actinomycetales
g__Streptococcus|9
g__Clostridium|6409
g__Sutterella
o__Clostridiales
f__Enterobacteriaceae|2516
g__Clostridium|26
g__Prevotella
f__Lachnospiraceae|488
f__Clostridiaceae|4513
g__Streptococcus
